# Modeling the Mental Lexicon as Part of Long-Term and Working Memory and Simulating Lexical Access in a Naming Task Including Semantic and Phonological Cues

**DOI:** 10.3389/fpsyg.2020.01594

**Published:** 2020-07-09

**Authors:** Catharina Marie Stille, Trevor Bekolay, Peter Blouw, Bernd J. Kröger

**Affiliations:** ^1^Department for Phoniatrics, Pedaudiology, and Communication Disorders, Faculty of Medicine, RWTH Aachen University, Aachen, Germany; ^2^Applied Brain Research, Waterloo, ON, Canada; ^3^Centre for Theoretical Neuroscience, University of Waterloo, Waterloo, ON, Canada

**Keywords:** neurocomputational model, spiking neural networks, computer simulations of natural language processing, behavioral testing, brain-behavior connection, semantic cues, phonological cues

## Abstract

**Background:**

To produce and understand words, humans access the mental lexicon. From a functional perspective, the long-term memory component of the mental lexicon is comprised of three levels: the concept level, the lemma level, and the phonological level. At each level, different kinds of word information are stored. Semantic as well as phonological cues can help to facilitate word access during a naming task, especially when neural dysfunctions are present. The processing corresponding to word access occurs in specific parts of working memory. Neural models for simulating speech processing help to uncover the complex relationships that exist between neural dysfunctions and corresponding behavioral patterns.

**Methods:**

The Neural Engineering Framework (NEF) and the Semantic Pointer Architecture (SPA) are used to develop a quantitative neural model of the mental lexicon and its access during speech processing. By simulating a picture-naming task (WWT 6-10), the influence of cues is investigated by introducing neural dysfunctions within the neural model at different levels of the mental lexicon.

**Results:**

First, the neural model is able to simulate the test behavior for normal children that exhibit no lexical dysfunction. Second, the model shows worse results in test performance as larger degrees of dysfunction are introduced. Third, if the severity of dysfunction is not too high, phonological and semantic cues are observed to lead to an increase in the number of correctly named words. Phonological cues are observed to be more effective than semantic cues.

**Conclusion:**

Our simulation results are in line with human experimental data. Specifically, phonological cues seem not only to activate phonologically similar items within the phonological level. Moreover, phonological cues support higher-level processing during access of the mental lexicon. Thus, the neural model introduced in this paper offers a promising approach to modeling the mental lexicon, and to incorporating the mental lexicon into a complex model of language processing.

## Introduction

### Normal and Disordered Speech

Speech processing involves complex cognitive, motor, and sensory processes. The cognitive system involved in speech processing includes pragmatic, semantic, syntactic and phonological components, and is linked with sensory and motor systems. Within this cognitive system, the *mental lexicon* serves as a basic knowledge repository for word forms and their meanings (long-term memory; Dell and O’Seaghdha, 1992; Levelt et al., 1999; Indefrey and Levelt, 2000; Elman, 2004). In many well-established models of language and speech production, the mental lexicon is implemented as a three-level neural network (Collins and Loftus, 1975; Garrett, 1980; Stemberger, 1985; Dell, 1986; Butterworth, 1989; Levelt, 1989; Caramazza, 1997; Dell et al., 1997; Levelt et al., 1999; Indefrey and Levelt, 2004; Indefrey, 2011). The concept network (in some models seen as a knowledge repository, located above the mental lexicon) stores and organizes the meanings of words. For example, the concepts corresponding to “dog” and “cat” are both represented as animals and thus are more closely associated with each other than the concepts corresponding to “dog” and “table.” A lemma network stores information about language-specific grammatical status of particular words. For example, “dog” is a *noun* and *singular* and is associated with morphological variants such as the plural form “dogs.” A phonological network stores sound sequences for words, such as the sequence of phonemes/d/, /O:/, /g/ (sound symbols are given here in SAMPA notation, SAMPA, 2005), and associates similar sounding words and syllables with one another. For effective speech processing to occur, the mental lexicon must include sufficient information at each level, and these levels must be appropriately associated with one another (e.g., the concept “dog” should be associated with the lemma “dog” and the lemma “dog” should be associated with the phoneme sequence /d/, /O:/ and /g/).

Speech and language processing occurs within two main pathways corresponding to speech production and speech perception. In both pathways, it is necessary to activate and to retrieve relevant information from the mental lexicon (a form of long-term memory) into working memory (Vitevitch et al., 2012). Working and long-term memory play an important role in speech and language processing. Working memory refers to the system that is assumed to play the role of keeping currently important things in mind during the performance of tasks like reasoning, comprehension, and learning (Baddeley, 2010). Information processed in the working memory can later be stored in long-term memory. Whether information is stored depends on several factors such as attention and the importance of the information. All representations of previously learned words are stored in long-term memory via the mental lexicon and can be transferred to working memory if these words are used during thinking or speaking (Jacquemot and Scott, 2006). During speech production, concepts are activated for a planned utterance, and associated lemmas and phonological forms are subsequently activated and then retrieved from different levels of the mental lexicon. Motor plans are then activated and retrieved from a second system called the mental syllabary (Cholin, 2008; Brendel et al., 2011; Kröger and Cao, 2015). This mental syllabary or mental action repository contains sensory and motor information used during speech articulation and needed for articulatory feedback (in its strict definition, the mental syllabary contains motor information exclusively, while the mental action repository includes sensory information as well, see Kröger et al., 2019). During speech perception, the mental syllabary and mental lexicon are used for identifying speech sounds, syllables, and words from the acoustic speech signal, i.e., for activating the appropriate phonological forms, concepts in order to be able to understand the meaning and the intention of a currently produced utterance.

In order to understand how a word is produced or understood correctly, easily and quickly, we have to describe the organization of the mental lexicon in more detail. It is known that at all levels within the mental lexicon, more than one item may be activated at one point in time. However, these items are often activated to different degrees. This results from the fact that the entries within the different levels of the mental lexicon are associated with each other through neural connections.

Associations at the concept level are based on semantic similarity. Associations occur not only with respect to categories (like “animal” or “object in a room”) but also with respect to more specific attributes such as size, shape, and color (McGregor and Waxman, 1998). Associations can be different from subject to subject depending on differences in personal experience both during and after speech acquisition. In general, associations are built up within the concept level on the basis of features like “has four legs,” which establish similarity relations between concepts like “dog” and “cat” as well as between the concept level and the word or lemma level, since each lexical entry is directly linked to one or more concepts (Lucas. 2000). In short, semantically related concepts are more strongly associated with one another than unrelated concepts.

Speech sounds, syllable constituents, and syllables are represented in the phonological level of the mental lexicon. Like at the concept level, the similarity between phonological items is based on neural associations of varying strengths. Associations at the lexeme level accordingly define similarity relationships between phonemes and syllables. For example, /cup/ and /cut/ would share more associations than /cup/ and /kale/ since they are more phonologically to one another (Levelt et al., 1999). Both in the concept level and in the phonological level, it can be assumed that the associations divide the respective levels internally and thus create superficial and deep networks (Kröger et al., 2016). In addition to these within level connections, a further type of connection exists between both the concept and lemma levels and the lemma and phonological levels, such that a concept (or lemma) representation is able to directly activate its corresponding lemma (or phonological) form. Overall, entries are linked within and between the different levels of the mental lexicon by learned associations. This network structure and the fact that the levels are interconnected allows for spreading activation between entries both within and across levels of the mental lexicon (Collins and Loftus, 1975).

Due to constrained or reduced activation spread, naming difficulties can arise (Foygel and Dell, 2000; Indefrey, 2011). Naming difficulties occur for patients with aphasia and for children with speech specific language impairments (SLI; Brackenbury and Pye, 2005; aphasia: Bragard and Schelstraete, 2007; Meteyard and Bose, 2018). The different levels within the mental lexicon correspond to different underlying causes of naming difficulties in SLI, as summarized by Best (2005). These include (i) impairments in storing semantic, lexical and phonological information; and (ii) impairments in accessing lexical-semantic or phonological form information for production, despite normally developed intellectual ability (Archibald and Gathercole, 2007). Evidence from aphasia indicates that naming deficits can result from a breakdown at different levels of word retrieval, most notably at the semantic and phonological levels (Martin and Laine, 2000). Leading researchers assume that in aphasia, lexical entries are more difficult to access but nonetheless still present in the mental lexicon (Jefferies and Lambon Ralph, 2005, 2006). Because of the similarity between symptoms of aphasia and SLI, and the procedures for their diagnoses, some researchers believe that it is possible to apply lessons from research on adults with acquired naming difficulties to develop a better understanding of SLI while taking the child’s stage of development into account (Best, 2005; Friedmann and Novogrodsky, 2008; Novogrodsky et al., 2010). A main difference, however, is that mental representations have been lost or are inaccessible in aphasia, and that they may have not yet been acquired, have been stored poorly or are inaccessible in SLI (McGregor and Appel, 2002; Brackenbury and Pye, 2005; Gray, 2005; Alt and Plante, 2006; Seiger-Gardner and Schwartz, 2008; Glück, 2011). Poor storage here refers to the storage of words and their meanings in the mental lexicon (Kail and Leonard, 1986; McGregor and Windsor, 1996). In the case of a pure retrieval disorder in SLI, the levels of the mental lexicon are intact, but the entries cannot be retrieved (Gershkoff-Stowe and Smith, 1997; Glück, 2011). These kinds of retrieval errors seem to be non-systematic and transient (Gershkoff-Stowe, 2002).

A common procedure for experimentally investigating the structure of the mental lexicon is drawn from the semantic or phonological priming paradigm. The semantic priming effect refers to the consistent observation that people respond faster to a target word (e.g., “cat”) when it is preceded by a semantically related prime (e.g., “dog”) rather than an unrelated prime (Lucas, 2000). In a similar way, phonological primes can be used. Phonological primes indicate that subjects are sensitive to phonological similarity and thus are aware of phonological and phonetic sound features (German, 2002). Moreover, priming effects produce information about the type of semantic or phonological neighborhood of a target item (e.g., semantic similarity concerning color, size or category; phonological similarity concerning a syllable initial consonant or concerning a vowel).

A related approach to investigating the structure and functioning of the mental lexicon involves the use of semantic and phonological cues in naming tasks. This is a technique to facilitate naming in naming tasks which are often used in language disorder diagnosis procedures as well as in speech and language therapies (Laine and Martin, 2006; Velez and Schwartz, 2010). In a picture-naming task, for example, cues are given if the subject provides an incorrect name or is unable to answer. A phonological cue often involves the oral presentation of the first sound of a target word (e.g., “b” for “bag; Abel et al., 2007). A semantic cue, on the other hand, typically involves an orally presented explanatory phrase like “there may be flowers in” for the target word “garden” (Abel et al., 2007). Upon presentation of these cues, semantic or phonological neighbors (or semantically or phonologically related features) are activated, which provides a further impulse within the word production process and can thus facilitate word production and correct naming (Nickels and Best, 1996; Brackenbury and Pye, 2005; Gershkoff-Stowe and Hahn, 2007; Velez and Schwartz, 2010; phonological cues: German, 2002).

Typical diagnostic tools that make use of picture naming with cues include the Word Range and Word Retrieval Test for 6- to 10-year-old German-speaking children (WWT 6-10; Wortschatz- und Wortfindungstest in German; Glück, 2011) and the Test of Word Finding (TWF; German, 2000) for English-speaking children. These tools are efficient for diagnosing language disorders because all core components of word production, including concept activation, lexical selection, and phonological code retrieval, are involved in picture naming (Indefrey, 2011).

A simple hypothesis is that phonological cues support the retrieval of phonological information for a target word, while semantic cues support the retrieval of semantic information for a target word (Meteyard and Bose, 2018). More specifically, it is hypothesized that phonological cues can help to overcome phonological impairments and semantic cues can help to overcome semantic impairments if the execution of a naming task including cues is seen as a learning procedure (e.g., Hickin et al., 2002; Van Hees et al., 2013). Furthermore, semantic cues are hypothesized to allow for the detection of semantic level dysfunctions, while phonological cues are hypothesized to allow for the detection of phonological level dysfunctions (Glück, 2011). As such, cues are used to identify lexical dysfunction within the word production process.

Cues are also used to differentiate between storage and accessing disorders. If cues are ineffective, it is an indication that certain mental representations are lost or have not yet been acquired (McGregor and Appel, 2002; Gray, 2005; Alt and Plante, 2006). If cues are effective, it is an indication that the mental representations have momentarily been disrupted due to an access disorder of some kind (Gershkoff-Stowe and Smith, 1997; Abel et al., 2007).

Previous studies indicate that both semantic and phonological cues can help to specify a semantic target for a picture (Li and Williams, 1991; Stimley and Noll, 1991; Meteyard and Bose, 2018). However, phonological cues seem to be effective for more individuals (Li and Williams, 1991; Lorenz and Ziegler, 2009; Van Hees et al., 2013). Thus, in picture naming, phonological cues appear to be more useful than semantic cues (Meteyard and Bose, 2018). While this observation applies to patients with aphasia, similar observations are found for children with SLI. McGregor and Windsor (1996) show that semantic and lexical cues in picture naming tasks reduce the error rate in children with word-finding deficits. McGregor (1997) shows that semantic errors are reduced by phonological cues, and similar results were found in German (2002) who describes an improvement in naming through a phonological therapy approach based on cues that are “phonological neighbors” of target words. The author suggests in this case that semantic errors arise from a breakdown at the phonological level. However, it remains unclear whether the phonological cues facilitate naming in case of a breakdown at the semantic or phonological level, as the cause of semantic errors is based only on hypotheses.

Therefore, questions remain about how different cue types affect naming performance in the context of neural dysfunctions at different levels of the mental lexicon. To investigate these questions, we introduce a large-scale neural model of the mental lexicon that is capable of simulating naming tasks. The structure of the mental lexicon in this model is based on the three-level approach described above, and simulations of naming tasks can include both semantic and phonological cues.

It is the goal of this study to use naming tasks to analyze the effects of different dysfunctions within and between different levels of our model and to measure the effect of semantic and phonological cues. This will allow us to associate specific *neural deficits (microscopic neural dysfunctions)* with *behavioral deficits (macroscopic lexical dysfunctions)* here in the case of a naming task with and without cues.

### Description of the Used Naming Task

The naming task we use is the *Word Range and Word Retrieval Test (WWT 6-10*; Glück, 2011*).* The WWT 6-10 is a standardized test for measuring (i) the size of the vocabulary stored in the mental lexicon along with word retrieval using this vocabulary (*RwO* in the following), (ii) the stability of word production, (iii) the increase in correct word production as a result of semantic cues (*RwS* in the following) and phonological cues (*RwP* in the following), and (iv) word comprehension as measured by pointing to the visual representation of a word (i.e., perception without production; Glück, 2011). The test is administered to children from 5 years and 6 months old to 10 years and 11 months old.

The test is made up of 95 target words subdivided into (i) 26 nouns which are visually presented to the subjects using one picture each (e.g., “wheelbarrow,” “crutch”), (ii) 23 verbs similarly presented (e.g., “to push,” “to wave”), (iii) 23 nouns for superordinate categories (e.g., “furniture,” “insects”) where each superordinate is activated on the basis of four pictures which present four different objects belonging to the superordinate target word (e.g., pictures of “bug,” “butterfly,” “bee,” and “grasshopper” for the target word “insects”), and (iv) 23 antonyms (opposites) for adjectives or adverbs (e.g., “old”) identified by a verbally presented word (e.g., “old” should be named if “new” is said by the test coordinator). The test is performed three times in direct temporal succession. First, the 95 target items must be named to measure the size of the vocabulary. Then, an exact repetition of this naming task is performed to measure the stability of the entries. Afterward, those items which were not produced correctly in either of the preceding runs are retested with semantic and phonological cues in order to measure the ability to facilitate word naming. In the case that the target word is a noun, these additional semantic cues correspond to (i) superordinate items, (ii) words representing concepts of additional characteristics such as the material from which the target concept is made of or its usage, and (iii) words representing locations where the item corresponding to the target word can be found. In the case that the target word is a verb, the cues are (i) a description of what happens during the action represented by the verb, (ii) a description of what can be achieved by that action, and (iii) a description of where that action takes place or how it is caused. In the case that the target word is an adjective or adverb, the cues are examples of objects or actions which exhibit the property described by the adjective or adverb. In the case that the target word corresponds to a superordinate concept, the cues are (i) example hyponyms that describe where and for what the superordinate is used, or (ii) describe details concerning the material composition of the concept represented by the superordinate or its location. Phonological cues are always the beginning of the phonological form (first sound) of the word. If the phonological form starts with a plosive or with the sound /h/, then the first two sounds are given. For example, in the case of “Ferse” /fEAze/, /f/ is sufficient as a phonological cue. In the case of “Berufe” /beru:fe/ or “Henkel” /hENkel/, the first two sounds are given: /be/ or /hE/ (sound symbols are given here in SAMPA notation, SAMPA, 2005). Finally, word comprehension is measured: after a verbal request, the children have to point out the mentioned item from a selection of four pictures.

Test results from the WWT can be compared with the standard test data (norm data) derived by Glück (2011). This data provides information on whether the results obtained are pathological or in the normal range. The norm data of WWT 6-10 are based on the results of 880 German children of appropriate age. However, norm data are only available for the WWT naming task (separate norms, *t*-values and percentile ranks for nine age groups from 5 years 6 months to 10 years 11 months), the WWT comprehension task (percentile ranks for nine separated age groups from 5 years 6 months to 10 years 11 months). Response times were also measured and published.

### The Used Modeling Approach

The neurocomputational model for simulating speech processing in this study is based on the NEF (Neural Engineering Framework; Eliasmith and Anderson, 2003; Eliasmith, 2013) and the SPA (Semantic Pointer Architecture, Eliasmith, 2013; Stewart and Eliasmith, 2014). This combination of the NEF and SPA allows for the implementation of cognitive as well as sensory input and motor output modules. We focus on implementing a mental lexicon model as part of long-term memory, along with specified working memories which are directly related to the three different levels of the mental lexicon. These parts of the overall working memory are used for processing speech units (concepts, lemmas, phonological forms) for a short period of time. Furthermore, the NEF and SPA allow for the simulation of behavioral tasks and thus enable a detailed approach for modeling the timing and the temporal sequencing of speech actions Simulations of visual digit recognition, question answering, memorizing of digits, etc., were done by Eliasmith et al. (2012). Related models of speech tasks like picture naming and word recognition were implemented by Kröger et al. (2016) and by Stille et al. (2019). The simulation of syllable repetition tasks in the context of the reduced dopamine levels, characteristic of some kinds of pathological speech was done by Senft et al. (2016). An initial simulation of word naming in case of neural dysfunctions at the lexical level was done by Stille et al. (2019). Neural models can, therefore, help address research questions regarding the relationship between low-level properties of neural systems and high-level linguistic behavioral patterns (Stille et al., 2019).

The NEF is based on three principles that provide a comprehensive mathematical framework for modeling spiking neurons and neural networks. These principles concern representation, transformation, and dynamics (Eliasmith and Anderson, 2003). The principle of representation enables spiking neurons to encode inputs (e.g., a visual signal of a letter, an acoustic speech signal of a syllable or word), outputs (e.g., a limb movement or a speech articulator movement) and internal representations (e.g., concepts, lemmas, phonological forms) into patterns of neural activity that can then be decoded to retrieve their meaning. To encode vectors of real values (e.g., the intensity of an auditory input or the output degree of muscular tension), groups of neurons, called ensembles, are used. Higher-level information (e.g., the frequency-amplitude spectrum of a complex sound, the control patterns of a complex movement or concepts, lemmata, and phonological forms) is often stored across more than one ensemble, i.e., in groups of neuron ensembles, called neural state buffers. Each neural ensemble consists of a specific number of individual neurons and each neural buffer comprises a specific number of neural ensembles. The neuron model conventionally used in the NEF and SPA is the leaky integrate-and-fire (LIF) neuron model. This neuron model provides a good balance between computational simplicity and neurobiological realism.

In contrast to a localist and connectionist approach to neural modeling, in which neurons or nodes are taken to represent the mean activation of a specific item (concept, lemma, or phonological form) at a specific point in time and in which only a simple rule for summarizing input activity and in which only a simple rule for calculating the output activity of a node is used, the integrate-and-fire approach is a more complex and biologically more realistic. First, the localist approach does not model individual neurons plausibly, since “nodes” and their “connections” are comparable to ensembles of neurons and connections between these ensembles in the NEF and SPA context. A leaky-integrate-and-fire neuron is also biologically more realistic than a localist “node”: incoming spikes are integrated over time in order to calculate the neuron cell membrane potential. New spikes are generated by neuron if the membrane potential exceeds a specific threshold value. This leads to the communication of spikes to all connected “downstream” neurons. A leak is added to the neuron model to reflect the fact that the cell membrane potential always decreases slightly over time and thus does not hold the potential generated by an incoming spike indefinitely.

The principle of transformation concerns how neural representations are transferred from one buffer or ensemble to another by neural connections. For example, a typical realization of neural connections between buffers is the transformation for states from a concept buffer to a lemma buffer and or the transformation of states from the lemma to the phonological form buffer. The neural connection between those two buffers contains the “knowledge” that implements, for instance, the transformation of the lemma “dog” into a specific phonological form. In the SPA, transformations are often implemented as associative memories (Voelker et al., 2014; Crawford et al., 2016).

In connectionist approaches as well as in our NEF-SPA approach transformations are implemented in the form of weight matrices which characterize the connection between two neural buffers. The most often used transformation is that from one set of N activations representing different items (called S-pointers; see below) to a second set of N S-pointers. Such a transformation is realized by connecting each neuron in one buffer to each neuron in the other. Intermediate buffers may be included to perform more complicated transformations.

A special subtype of associative memory is the *cleanup memory* which is needed if the result of a transformation is unclear, such that more than one item is activated in the output buffer. A cleanup memory helps to select a “winner” item from amongst those that are activated (Crawford et al., 2016). While in a transformation one set of N S-pointers is mapped to a second set of N S-pointers, in a cleanup memory a set of N S-pointers is associated on the same set of N S-pointers, such that each S-pointer is associated with itself. Because the cleanup memory buffer “knows” the clean S-pointer, or the ideal neural representation of a particular item, a nearby-S-pointer (a non-clean representation of that item) now can be identified as representing exactly that item by replacing this non-clean S-pointer by its clean prototype. This prototype will be selected from the cleanup memory. Thus, all items represented by non-clean S-pointers provided as input to a cleanup memory are replaced by the prototypical S-pointer representations of these items at the output of the cleanup memory.

More mathematical details concerning how information represented and transformed using neural buffers, can be found in Eliasmith (2013).

The principle of neural dynamics concerns how activity patterns occurring in neural ensembles to change over time, either due to changes in input to the ensemble or due to recurrent connections within the ensemble. With recurrent connections, ensembles or buffers can implement working memories for values or states (Eliasmith, 2012). Such working memories are an important part of the present model because they can maintain information about cognitive units (concepts, lemmas, phonological forms) for a short period of time and thus enable language processing.

The SPA (Stewart, 2012; Eliasmith, 2013) builds on the NEF to allow the modeling of complex cognitive processes by grouping neural ensembles in sophisticated functional units (Eliasmith et al., 2012, 2016) controlled by a central executive system (Stewart T. C. et al., 2012; Stewart T. et al., 2012). The central executive system of a SPA model is called the *task control module* and designed to emulate the basal ganglia-thalamus-cortex loop for cognitive action selection (Stewart et al., 2010a; Stewart T. C. et al.,2012). The main items processed by the SPA are S-pointers. These S-pointers are high-dimensional vectors referring to a cognitive item or to a complex input or output signal. S-pointers can be interpreted on the one hand as “representations” from a cognitive viewpoint and can be associated on the other hand with specific neural activity patterns, occurring in specific neural buffers. Thus, a specific neural activity pattern in a buffer at a given moment in time can be interpreted either as a mathematical vector of values encoded by spiking neurons, or as a specific sensory, motor, or cognitive state. At the cognitive level, S-pointers therefore allow one to interpret neural activity in terms of symbol-like representations. Furthermore, since the activity pattern in a buffer changes over time, a buffer can represent sequences of different S-pointers over time.

S-pointers are also vehicles for representing *actions* like “start the production of a word” (‘SPEAK’) or “select a noun” (‘PRODUCE_NOUN’). These action S-pointers are activated in a task control buffer and are associated with rule-like effects in the task control module composed of basal ganglia and thalamus models (Stewart et al., 2010a, b). These rule-like effects disinhibit ensembles of neurons associated with specific actions in the thalamus when the input to the basal ganglia is sufficiently similar to a specific S-pointer or a combination of S-pointers. The task control buffer and basal-ganglia-thalamus complex are interconnected, forming a cortical action selection loop (ibid.). This loop allows for S-pointers to produce motor actions or cognitive actions.

With respect to the control of a whole NEF-SPA model, S-pointers appearing in an input control buffer trigger action selection in the task control module including basal ganglia and thalamus. Here, the cortex-basal ganglia network evaluates which action is the most useful action at a given point in time. All possible actions which can be performed by the model are encoded as S-pointers. The dot products between S-pointers specifying all possible actions and the S-pointers specifying the current situation are calculated. The action S-pointer leading corresponding to the highest dot product is then selected and the associated action is then executed.

In the context of the WWT task, four different questions or orders are given by the test supervisor: “what is this?” in order to evoke the naming of a noun, “what is he/she/it doing?” in order to evoke the naming of a verb, “what is the opposite of…?” in order to evoke the naming of an adjective/adverb, and “what is this all together?” in order to evoke the naming of a superordinate. These different conditions are coded by different S-pointers in the input buffer if the WWT task demands it.

In addition, the NEF-SPA approach includes temporal modeling of neural activation. At the level of buffers, input activation can change over time (e.g., S-pointer A1 is activated using a step function over a specific time interval t1-t2 because in that time interval the test supervisor gives advice) and leads to temporal variations in S-pointer activation at subsequently connected buffers (see below for our concrete NEF-SPA model). It can be seen that the temporal dynamics of a step-function input always includes a specific rise time because the generation of spikes encoding the represented value takes time. Overall, timing is controlled by the neuron model parameters along with variations in the input signals which are processed by the task control module of our model. The timing of these input control signals is based on the task under execution as specified by WWT.

Neural models based on the NEF and SPA approach are implemented using a Python-based library called Nengo (Bekolay et al., 2014; Sharma et al., 2016). In Nengo, high-level commands are available for configuring and running a model comprised of neuron ensembles, neuron buffers, and connections between these ensembles or buffers. The implementation of the task control module (central executive) can be realized easily in this framework by defining all actions in the form of semantic pointers and by providing this information to the neuron ensembles and buffers defining a model of the basal ganglia and thalamus (Stewart et al., 2018). Our model for simulating the WWT is written in Nengo and the source code is available (see below).

## Materials and Methods

### The Computer-Implemented Model

**FIGURE 1:**
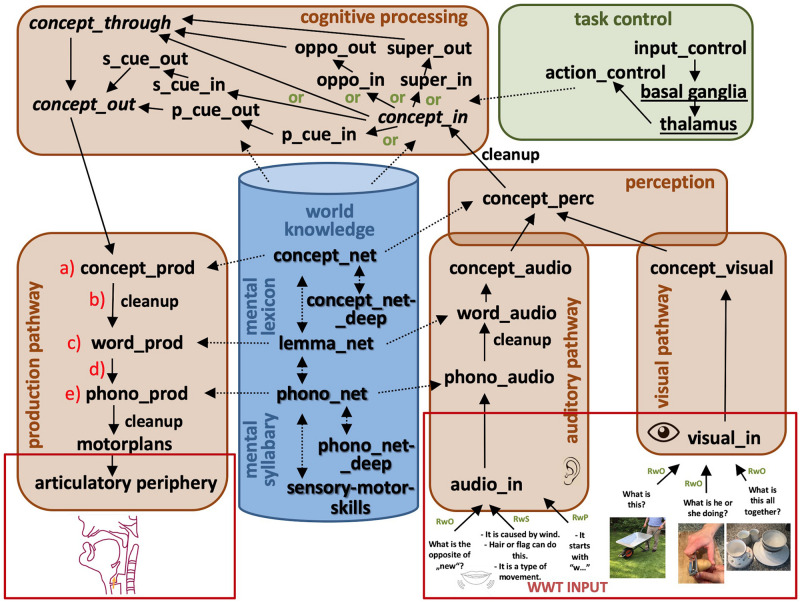
Modules (cognitive processing, task control, production, and perception pathways) and long-term memory components (world knowledge: mental lexicon and mental syllabary) of our large-scale neural model. Arrows indicate neural connections between buffers. Buffers within the perception and production pathways allow neural encodings (i.e., neural activation patterns) of S-pointers defined in the mental lexicon and mental syllabary (dashed arrows). S-pointer activity is passed from one buffer to the next within pathways and modules as well as between modules (normal arrows). Short-term memories (recursive buffers) are marked by cursive letters while all other non-cursive black colored words label non-recursive buffers. Associative memories including cleanup memories are marked by an extra word attached to the arrow. Different gateways (see green marked the word “or”) are controlled by the task control module. The underlined words within the task control module represent specific neural submodules like basal ganglia and thalamus. (a–e): indicate buffers or associative memories in which neural dysfunctions are introduced by changing the level of ablation in a specific buffer or associative memory.

Adopting the architecture of well-known models like those of Dell and O’Seaghdha (1992) and Levelt et al. (1999) our model comprises an auditory and visual input pathway as well as production and articulatory output pathway, both of which are tightly connected with small-capacity working memory buffers representing the different levels of the mental lexicon. The mental lexicon itself is part of long-term memory. Thus, in the input pathway, an auditory signal is transformed into a phonological form, a lemma, and a concept. Visual input is directly transformed into a concept. In the output pathway, a concept is transformed into a lemma and a phonological form before motor plans are activated to produce speech. The architecture for our neural model for simulating parts of the WWT 6-10 for naming without cues (RwO), naming with semantic cues (RwS), and with phonological cues (RwP) is given in Figure 1. The model is comprised of seven modules: (1) a visual perception pathway module, (2) an auditory perception pathway module, (3) an overall perception module, (4) a production and articulation pathway module, (5) a cognitive processing module, (6) a task control module and (7) a knowledge repository module consisting of the mental lexicon and mental syllabary (Figure 1). The overall perception module is included because both the auditory and visual pathways are processing mainly words, and thus both pathways end with a similar semantic representation of words. The production pathway, on the other hand, always starts with a semantic representation of a word. Thus, it seems to be straightforward that the cognitive processing pertaining to the selection of words is tightly connected to the concept level. Therefore, the cognitive processing module in the model is tightly connected to concept buffers representing both the ending of the perception pathway as well as the beginning of the production pathway. The organization of the cortical buffers within the task control module and their connection with the basal ganglia and thalamus networks is defined by the NEF-SPA approach itself (Eliasmith, 2012). This task control module is tightly connected to the cognitive processing module in our model because the action S-pointers activated by the task control module define the concrete neural pathway chosen within the cognitive processing module. These pathways all start with neural activation patterns occurring in the *concept_in* buffer and end with neural activation patterns occurring in the *concept_out* buffer. However, the concrete pathways that are chosen in the cognitive processing module for the selection of nouns, verbs, adjectives/adverbs, and superordinates are different (see Figure 1). Architectures like the one developed for the WWT task can differ slightly from task to task because the architecture never represents the whole brain but only those parts of the brain involved in the execution of the specific task which is modeled.

In older connectionist approaches (Dell and O’Seaghdha, 1992; Levelt et al., 1999) neural activation are encoded in a localist manner. Our model, based on the NEF and SPA (Eliasmith, 2013) using leaky-integrate-and-fire neurons, is more specific in a biological sense. The activation of a concept, for example, is modeled by a distributed neural activation pattern of all neurons within a neural state buffer. Thus, different activation patterns within a neural buffer are able to represent different concepts in a concept buffer, different lemmas in a lemma buffer, and different phonological forms in a phonological form buffer.

The model is designed with respect to current knowledge of the interaction between components of the mental lexicon and speech processing modules. Thus, the following hypotheses are taken as a basis for designing the model: In order to model all neural parts of the brain involved in a naming task, we need (1) an input control buffer for defining the timing of basic events like the occurrence of an order given by the test supervisor, or the initiation of an answering or reaction procedure on the part of the participant. (2) Triggering an action is done if a new task control S-pointer is activated in the input control buffer. This leads to action selection and thus to the activation of a specific action within the action control buffer. The input control buffer reflects the timing of each interaction between the test supervisor and test subject (i.e., the model). (3) Perceptual input is processed by the perceptual pathway. Here only the visual and the auditory pathways are included in our model. Both pathways end with cognitive representations of words, stimulated mainly by pictures in the WWT. (4) The processing of auditory input leads to the activation of different levels of the mental lexicon. (5) The pathways within the cognitive processing module are modeled simply with respect to the WWT task. Each concept needs to be processed with respect to the word category and thus with respect to different action control commands generated in the task control module. (6) The production and articulation pathway is modeled straightforwardly as well. A concept activates a lemma and the lemma activates a phonological form that subsequently activates motor plans for each syllable controlling the articulatory execution.

The priming commands concerning the WWT task are encoded at the input control level within the task control module. Thus, this module controls the overall dynamics for all modules, and is coupled to the timing of the visual and auditory input. The cognitive processing module processes concepts which stem from the highest level of the perception module and produces a representation that is forwarded into the production and articulation pathway.

The knowledge repository (long-term memory; blue in Figure 1) consists of the mental lexicon and mental syllabary. Within the mental lexicon, S-pointers are defined for the 95 target words of the WWT 6-10 and for three to four visually related concepts for each presented picture (Kröger et al., 2016; Stille et al., 2019). These visually related concepts are derived from the WWT photo templates. Examples are shown in Figure 1 below right. For the example in the middle with the target item “to peel,” visually related concepts are “peeler,” “hand,” and “potatoes.” In addition, a set of semantic and phonological relations and the related concepts, words, and phonological forms are included for each of the 95 entries so hat semantic and phonological cues are able to help to activate the naming of each target word. In total, the mental lexicon comprises 1204 concepts as well as their related S-pointers. Moreover, lemmata and phonological forms, as well as their related S-pointers, are defined for all 1204 concepts. Thus, a semantic, as well as a phonological, S-pointer network has been defined which is able to model all introduced semantic as well as phonological relations. The S-pointer relations between the concept, lemmata, and phonological form levels are stored using associative memories. These relations are one-to-one-associations between a concept form, a lemma form, and a phonological form for a given word. The relations between words occurring within the semantic S-pointer networks are implemented by naming all relations between concepts stored in a *concept_net* layer and concepts stored in a *concept_net_deep* layer using a category-relation-operator called “assoc_with,” which, for example, relates concrete objects like “car,” “bus,” and “bicycle” to a category item like “objects for transportation of one or more humans.” Once these relations between S-pointers are defined, a complete S-pointer network is developed and S-pointers that are related to each other by a category-relation-operator are realized as similar S-pointers with a large dot product (Crawford et al., 2016). By differentiating between these two levels within the semantic layer, semantic relations can be defined not just for objects, but also for actions like “to peel,” and “to open,” which are both “movements” for “food preparation” (see Table 1 for more examples). The concept network within the concept level of the mental lexicon includes all acquired concepts and their relations or associations. A within-level concept association might be “plant” (object) “is associated with” (assoc_with) “invertebrate” where “plant” is a part of the concept_net and “invertebrate” a part of the concept_net_deep (cf. Table 1). In a similar way, the phonological S-pointer network stores specific relations between phonological forms by associating phonological forms stored in the *phono_net* layer with phonological forms stored in the *phono_net_deep* layer. In the same way that superordinate concepts for different entries are stored within the concept_deep_net (e.g., “animals” for concepts like “dog,” “cat,” or “mouse”), superordinate phonological forms (e.g., for two forms which have the initial sound in common, e.g., /SEE/ for phonological forms like /’Pw_St_SEE_len/ (the German word “to peel”) and /’Pw_St_SEE_re/ (the German word “scissors”) are stored within the deep phonological network (phono_deep_net, cf. Tables 1, 2; Kröger et al., 2016). Thus, concept-level associations are organized between concepts within two concept layers, while phonological associations are organized between phonological forms stored within two phonological form layers (Figure 1). For more information on how S-pointer networks are organized and implemented, see Blouw et al. (2016), Crawford et al. (2016), and Kröger et al. (2016). Besides this within-level association, the model incorporates between-level associations. Thus, each concept is associated with one item at the lemma level and each lemma item is associated with a phonological form. Words or lemmata realize the middle level of the mental lexicon and specify grammatical attributes (noun, verb, gender, number, etc.) while phonological forms realize the lowest level of the mental lexicon.

**TABLE 1:** The modeling of within level associations at the concept level within the mental lexicon given in extracts for one example, i.e., some words within the concept level are given here which are associated with deep concepts: deep concepts need not be real words in the target language (e.g., “FoodPreparation”).

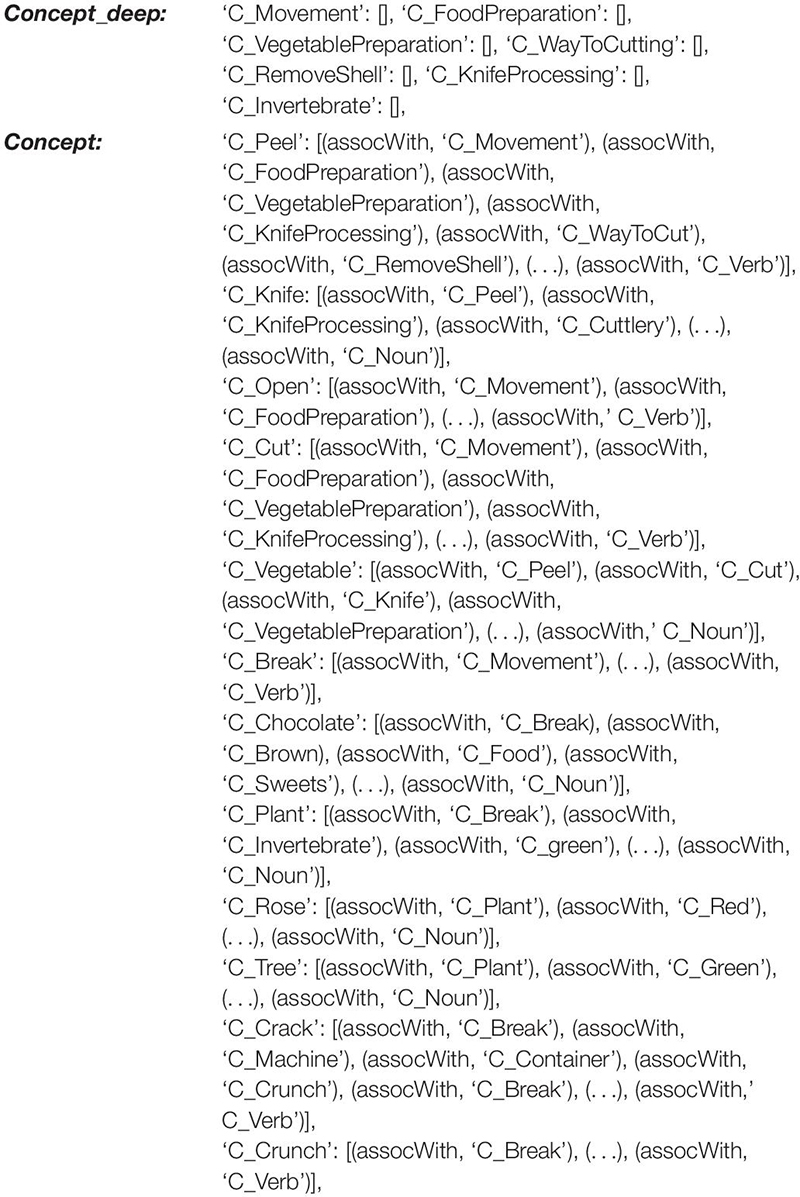

*Thus, the concept level includes two layers: a surface layer and a deep layer.*

**TABLE 2:** The modeling of within level associations at the phonological level within the mental lexicon is given here in detail for some phonological forms which are associated within the phonological S-pointer network with the deep phonological forms.

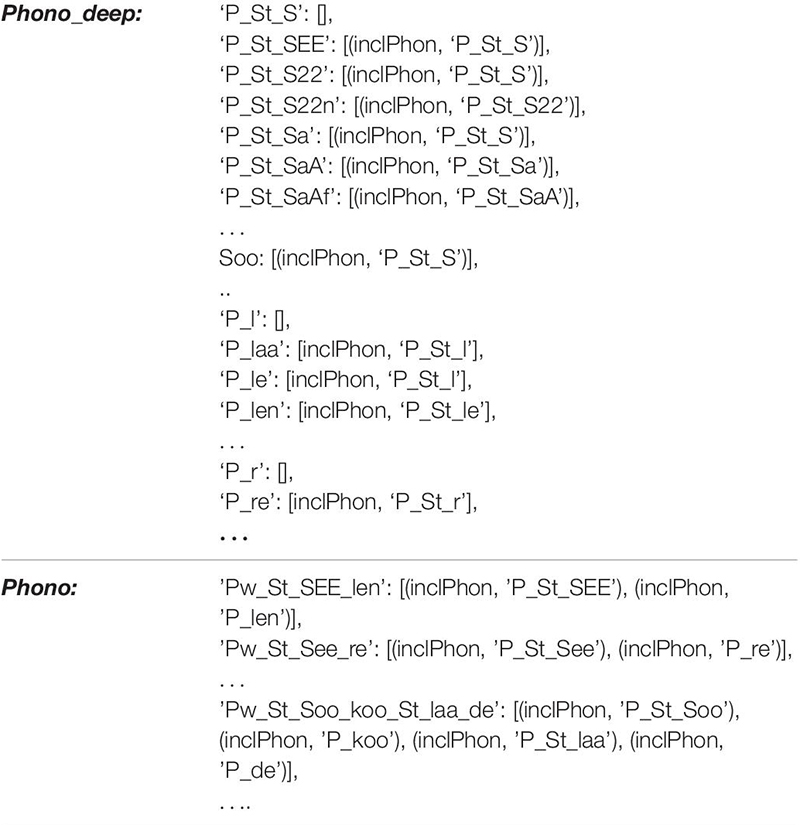

*“P_” means phonological form; “St_” means stressed syllable; “Pw_” means phonological form of a whole word; the following letters indicate the phonological transcription of the word or syllable or part of the syllable. The German words which are associated here by the deep form, e.g., “SEE” are “schaelen” (to peel) and “Schere” (scissors). The phonological level is comprised of two layers: the deep layer and the surface layer, which interact with each other. Seen in more detail, the phono_deep layer itself splits into different layers. For example, /S22n/ refers to /S22/; and /S22/ refers to /S/.*

Thus, two hierarchically organized S-pointer networks arise in our model: A semantic S-pointer network and a phonological S-pointer network (Kröger et al., 2016). The concept_net and phono_net can be called surface network parts. The surface conceptual network stores 1202 concepts that can be activated directly in concept buffers within the production or perception pathways, as well as in concept buffers within the cognitive processing module. These buffers are part of the working memory and are used for speech processing, particularly during word access. The deep conceptual network (*concept_deep* in Figure 1) stores 663 deep concepts. It includes all superordinate concepts which are needed for establishing the semantic relations within the conceptual network.

In the case of the phonological form S-pointer network, all phonological forms of words which can be activated in the lemma level are represented in the surface phonological network (1110 phonos; *phono_net*, Figure 1). All deep phonological forms such as syllables and subsyllabic structures (e.g., initial consonants or consonant clusters) are stored in the deep phonological network (1119 deep phonos; *phono_deep*, Figure 1 and Table 2).

Within-level associations contrast with between-level associations, which are implemented using associative memories. These associations involve a direct one-to-one mapping between the S-pointers represented in different state buffers (e.g., concept, lemma, and phonological form buffers). Associative memories, also called “connecting buffers,” are indicated by black arrows between two buffers in Figure 1.

In summary, the model consists of a knowledge repository, which is implemented as a long-term memory. 1202 concepts and 663 deep concepts are stored at the concept level. 1202 lemmata are stored at the lemma level. At the phonological level, 1110 phonological forms and 1119 deep phonological forms are stored. The concepts are connected to lemmas and associated word forms by connecting buffers (black arrows in Figure 1). The vocabulary size is not the full size of a child’s vocabulary, but only representative. The vocabulary used here was designed in order to be large enough to perform the two specific tasks of the WWT (naming with and without cues). Therefore, the present vocabulary can be interpreted as a basic vocabulary needed to perform the tasks successfully, but it is still large enough for mistakes to arise. Besides the basal ganglia-thalamus-complex, all other buffers in the model are working memories. These buffers are small-capacity working memories and are able to represent the current cognitive state by integrating a small amount of information over a brief period of time. The surface networks and the lemma network can be activated directly by the production or perception pathway buffers, as well as by concept buffers within the cognitive processing module (dashed errors in Figure 1). It should be noted that these buffers are only used for speech processing and word access. Learning processes have not been modeled; rather, neural connection weights are calculated to implement the defined associations between buffers (Eliasmith, 2013; Stewart and Eliasmith, 2014). Similar remarks apply to the implementation of task control for the individual tasks, which is predefined in a task-specific way (Stewart et al., 2010b; Stewart T. et al., 2012). Only the decision processes required for the two tasks under consideration were implemented.

Grammatical knowledge concerning the formation of sentences is still beyond the scope of our current model and thus no grammatical attributes are associated with the lemmata. However, lemmata are represented by S-pointers and can be activated in our model both in the production pathway and in the perception pathway (Figure 1). While a phonological representation is a low-level representation within the mental lexicon, it is also a high-level representation within the mental syllabary after syllabification. Thus, S-pointers of polysyllabic words cannot be directly converted in articulatory forms but need to activate a sequence of S-pointers representing monosyllables.

Input can be fed to the model via two perception pathways: the auditory pathway and the visual pathway (Figure 1). Within the auditory pathway, auditory input encoded in the neural buffer *audio_in* will activate the appropriate phonological form within the neural buffer *phono_audio*, a lemma within the buffer *word_audio*, and a concept within the neural buffer *concept_audio*. In the case of visual input, a concept within the buffer *concept_visual* will directly be activated if a visual input is encoded in the buffer *visual_in*. Because the lower-level auditory and visual pathways are not modeled in detail, our model cannot account for any behavioral deficits arising from deficits in visual and auditory processing; the model directly activates the phonological form for an occurring auditory input and the conceptual form of an occurring visual input.

Visual and auditory inputs are forwarded to the *concept_perc* buffer, which activiates the semantic knowledge stored in the concept S-pointer network of the mental lexicon (dashed arrow in Figure 1). Because of the coactivation of many associated concepts, a cleanup process is introduced as a part of the association between buffer *concept_perc* and buffer *concept_in*, or in other words as a step in the process of forwarding concepts from the perception pathway to the cognitive processing module. The cleanup process is modeled within an associative memory implemented between the *concept_perc* buffer of the perception module and the *concept_in* buffer of the cognitive processing module. Because associative memories are indicated by arrows between neural buffers in Figure 1, these arrows are labeled as cleanup in the case that the corresponding to cleanup memories.

The task control module is responsible for action selection and realizes the timing of all neural processes occurring within the model during any simulation. The temporal activation patterns occurring within the *input_control* buffer are shaped with respect to the temporal structure of the task under execution and thus represent the priming of the model with respect to the task that is currently being executed. In the case of simulating the WWT, the task control module differentiates amongst the four subtasks of (i) naming an object based on visual input, (ii) naming an action based on visual input, (iii) naming the opposite based on audio input, and (iv) naming the superordinate based on four visual inputs. For each of these subtasks occurring in each run of the WWT without cues, the control module switches the forwarding of the *concept_in* representation toward the *concept_through* buffer (case i and ii), toward the *oppo_in* buffer (case iii), or toward the *super_in* buffer (case iv). In the case of runs with cues, the path toward the *cue_in* buffers (*p_cue_in* or *s_cue_in*, depending on whether a phonological or a semantic cue is presented) is also opened.

**FIGURE 2:**
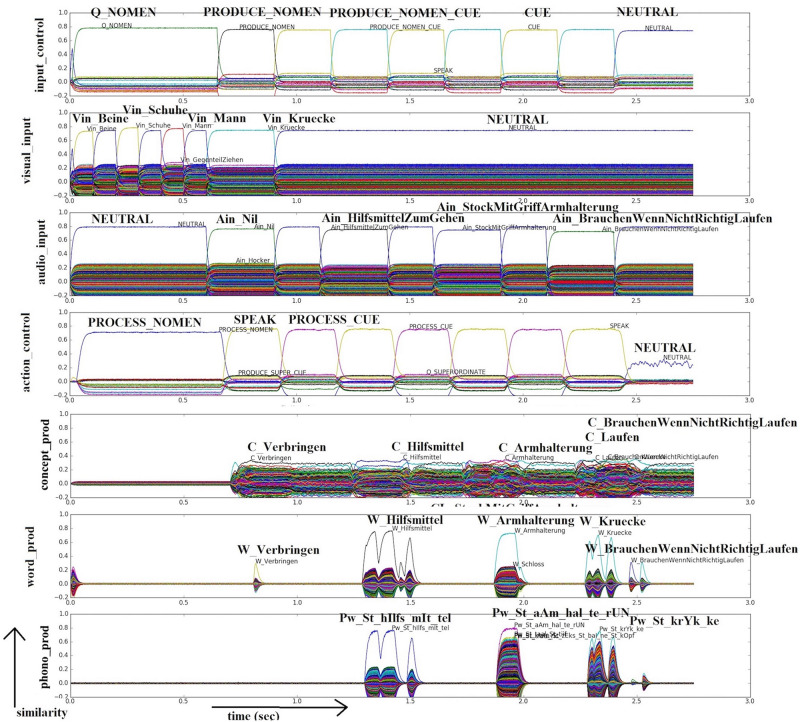
Similarity values of S-pointer activations occurring in different neuron buffers over time during simulation of a picture naming task (noun) based on visual input (Vin): “Kruecke” (“crutch”) and additional auditory input due to semantic cues (translated visual input: “legs”; “shoes”; “man”; “crutch”; translated audio input: “walking aid”; “stick with handle arm holder”; “need if not walking properly”). Rows indicate similarity values for the representations encoded in different neural state buffers over time (t). The different neural state buffers refer to the schematic model in Figure 1. Each S-pointer similarity value over time is represented by a trajectory with a specific color. The similarity value of an S-pointer at a point in time is the dot-product of that S-pointer with the encoded representation. The number of colors is limited, so the same color may occur for different S-pointers. Row 1: Input control buffer, row 2: visual input buffer, row 3: audio input buffer, rows 4–6: buffers for concepts, words, and phonological forms within the production pathway. The translated task in this simulation is ‘Q_NOUN‘; ‘PRODUCE_NOUN‘; ‘CUE‘; ‘PRODUCE_NOUN_CUE’ (translated *input_control* buffer), controlled by ‘PROCESS_NOUN‘; ‘SPEAK‘ and ‘PROCESS_CUE‘ (translated *action_control* buffer). The two lines represent different runs (RwO and RwS). In RwS there are semantic cues additively represented (see *audio_in*). The production of the word is displayed in the buffers *concept_prod* (translated output: “to spend”; “aid”; “arm holder”; “to walk”; “need if not walking properly”), *word_prod* (translated output: “to spend”; “aid”; “arm holder”; “crutch”; “need if not walking properly”), and *phono_prod* (translated output: “aid”; “arm holder”; “crutch”). In the buffer *phono_prod*, the target word is displayed in a phonetical form with the stressed syllable (phonetic transcription with SAMPA, 2005). All other buffers defined in the model are present but not shown in this figure for clarity.

**FIGURE 3:**
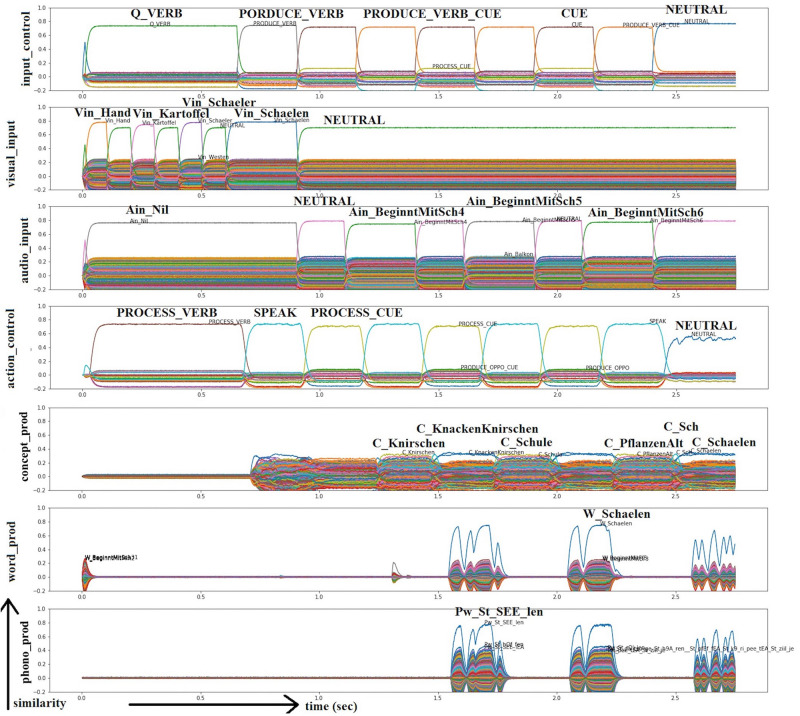
Similarity values of S-pointer activations occurring in different neuron buffers over time during simulation of a picture naming task (noun) based on visual input (Vin): “Schaelen” (“to peel”) and additional auditory input due to phonological cues (translated visual input: “hand”; “potatoes”; “peeler”; “to peel”; translated audio input: “starts with sch”). Rows indicate neural similarity values in different neural state buffers over time (t; see Figure caption 2). In this case, the translated task is ‘Q_VERB‘; ‘PRODUCE_VERB‘; ‘CUE‘; ‘PRODUCE_VERB_CUE’ (translated *input_control* buffer), controlled by ‘PROCESS_VERB‘; ‘SPEAK‘ and ‘PROCESS_CUE‘ (translated *action_control* buffer). The two lines represent different runs (RwO and RwP). RwO and RwP were included consecutively in one simulation. In RwP there are phonological cues additively represented (see *audio_input*). The production of the word is displayed in the buffers *concept_prod* (translated output: “crunch”; “crack crunch”; “school”; “plants old”; “to peel”), *word_prod* (translated output: “to peel”), and *phono_prod* (translated output: “to peel”). In the buffer *phono_prod*, the target word is displayed in a phonetical form with the stressed syllable (phonetic transcription with SAMPA, 2005). All other buffers defined in the model are present but not shown in this figure for clarity.

In the case of naming nouns (*n* = 26) and verbs (*n* = 23) shown in a picture, the test administrator asks, “what is this?” or “what is he/she doing?” To simulate this task, S-pointers associated with nouns or verbs are activated in the visual input buffer for time intervals which are assumed to correspond to the subject focusing on the presented picture. The concept activated in the visual input module is then directly passed through the visual pathway to the cognitive processing module (from *concept_in* buffer via and *concept_through* buffer to *concept_out* buffer) and passed to the production pathway module in order to articulate the resulting word. The verbal command of the administrator is not modeled in the auditory pathway. The noun and verb naming tasks are differentiated through the action-S-pointer that initiates the task in the *input_control* buffer within the task control module; for nouns, the S-pointer ‘Q_NOMEN’ is activated, while for verbs the S-pointer ‘Q_VERB’ is activated, followed by the ‘PRODUCE_NOMEN’ or ‘PRODUCE_VERB’ commands within this buffer (see row input_control in Figures 2, 3).

In the case of the task to produce an adjective or adverb (*n* = 23) that represents the opposite of the spoken adjective or adverb given by the administrator (e.g., “complicated” in the case of “simple”), the input word is given as audio input and thus word activates all parts of the mental lexicon as shown in the auditory pathway of Figure 1. The instruction sentence “what is the opposite of …?” is not given as audio input. Instead, as in all other subtasks, the model is provided a unique action-S-pointer representing the task, in this case the S-pointers ‘Q_OPPOSITE’ followed by ‘PRODUCE_OPPOSITE’ at the level of the *input_control* buffer. In the cognitive processing pathway, the concept input is routed to the *oppo_in* buffer. The opposite is generated by an associative memory between the *oppo_in* and *oppo_out* buffers (see Figure 1). This associative memory stores antonym relationships (opposite relationships) between the test words and their antonyms.

To produce superordinate concepts (*n* = 23) the subject must find and produce a noun which names the superordinate of four items presented visually (e.g., “furniture” for “chair,” “cabinet,” “bed,” and “couch”). Thus, the four items are encoded in the *visual_in* buffer of the visual perception pathway, forwarded as concept items to the cognitive processing module, and routed to the *super_in* buffer there. The superordinate is encoded in the *super_out* buffer (see Figure 1) using an associative memory that stores knowledge concerning concepts and their superordinates as implemented in the concept S-pointer network of the mental lexicon. The task is identified within the task control module by the action-S-pointer ‘Q_SUPERORDINATE’ and subsequently ‘PRODUCE_SUPER’ being encoded in the *input_control* buffer.

For runs of the WWT involving cues, three semantic or phonological cues are provided. All cues are included in the vocabulary of our neurocomputational model. Because semantic cues are expressed as phrases or as whole sentences (for example the phrase “hasLegs” in the case of the target concept “dog”), these “phrases” or “sentences” are modeled like target words by using only one S-pointer per cue. Parsing and encoding whole sentences is outside the scope of work presented in this paper. Typical whole-sentence-cues and whole-phrase-cues are given in Table 3.

**TABLE 3:** Cues used in WWT and for simulation.

		Semantic Cues in WWT			Modified Semantic Cues for Simulation			Phonological Cues in WWT and Simulation (German version)
				
Word Type	Sample word	First sem. cue	Second sem. cue	Third sem. cue	First sem. cue	Second sem. cue	Third sem. cue	
Noun	wheel-barrow	Kind of vehicle to push	To transport stuff	Mostly in the garden or construction side	push	transport	garden	Starts with “S…”
Verb	To peel	Way to cut	To remove shell	Need a knife for this	cut	shell	knife	Starts with “S…”
Adjective/Adverb	new/old	House that is just built is new	House that exists for years and is broken is…?	What is the opposite of new?	build	broken	old	Starts with “a…”
Super-ordinate	vegetable	This includes spinach, pees and, carrots.	For cooking, e.g., lunch	Grows in the garden, on the field or in the greenhouse.	spinach	cook	field	Starts with “ge…”

*Examples are given here for each type of WWT subtask.*

To process the cues within the cognitive processing module, a *concept_through* buffer is included, since in the case of additional semantic or phonological cues, a word candidate may be activated at the level of the *concept_through* buffer while a high level of activation at the *concept_out* level only occurs if additional cues are given via the path defined by the *cue_in* and *cue_out* buffers (see Figure 1).

The activation pattern of the *concept_out* buffer within the cognitive processing module starts to activate the production pathway by activating neural representations of concepts, lemmata, and phonological forms in the neural buffers *concept_prod* via *word_prod* down to *phono_prod*. For the association from the *concept_prod* buffer to the *word_prod* buffer, further cleanup processes are needed to map the most relevant semantic pointer from the concept production buffer to the word production buffer. Motor plans are not explicitly modeled. We use phonological forms for evaluating the outputs of the model during simulation experiments.

The outputs of the model are similarity plots that represent the information encoded by the neural activity of each buffer shown in the schematic model (Figure 1; Eliasmith, 2013). Similarity plots show the overall neural activity occurring within each buffer ordered with respect to all semantic pointers which are represented by this activity (see Figures 2, 3).

Our complete speech processing model (Figure 1) includes about 22 buffers and about 26 associative memories, of which 4 are cleanup memories. All semantic pointers are of 64 dimensions, and thus, each neuron buffer includes 64 ensembles. This dimensionality was determined to be optimal for picture naming tasks in previous experiments (Stille et al., 2019). Based on Nengo’s default settings, each ensemble consists of 50 neurons leading to 64 x 50 = 3250 neurons per buffer. Because associative memories contain twice as many neurons as normal buffers, the model contains 22 x 3250 = 71500 neurons for buffers and 26 x 6500 = 169000 neurons for associative memories (including cleanup memories). Each neuron ensemble within basal ganglia and thalamus consists of 50 neurons leading to 2100 neurons in basal ganglia and 400 neurons in the thalamus. All parameters concerning neurons and neural connections are set to the Nengo default values. The simulations were conducted on three Windows computers with Intel^®^ Core^TM^ i7 processors. For the WWT simulations that include five different settings of the neural model in order to model five different types of dysfunctions (see below), 15,675 simulations were performed. Adding up the simulation time on all three computers results in a total simulation time of 86 days, 23 h, and 3 min.

### Simulations

Two simulation experiments were performed. One experiment concerns to the naming task without (RwO) cues and the other experiment concerns naming with semantic (RwS) and phonological cues (RwP). In order to investigate the effect of cues, a third simulation is carried out as a control (RwT). In this simulation, the time span for the answer is extended so that it corresponds to the time variance with cues. In this way, the effect of the cues can be differentiated from a possible time effect. For the first experiment, three runs were performed (RwO_1-3). The simulated data is compared with the norm data of the WWT 6-10 the age group of 5;6 to 6;5-year-old children (Glück, 2011). The size of the vocabulary within the present mental lexicon is limited (see above, chapter 2.1). This vocabulary can be interpreted as a basic vocabulary that is needed by the mental lexicon in order to perform the tasks successfully. It is still large enough that errors can occur. Therefore, the age group of 5;6 to 6;5-year-old children is used for the present study since the size of the vocabulary is the smallest for this age category. The size of the present mental lexicon is only representative of the age category, but most suitable for comparison. The norm data indicate that children of this age correctly name a maximum of 76 items. On average, this age group can correctly name 39 out of a total of 95 items. Values between 27 and 52 correctly named items are within ± 1 standard deviation from the mean value (39). This is the area in which the performance is to be assessed as normal. The cut-off value is therefore 27 of correctly named items. All results involving fewer than 27 correctly named items are to be assessed as pathological. For the second experiment one run with semantic cues (RwS), one run for phonological cues (RwP), and one run for the control variable (RwT) are performed. These runs are meant to be compared statistically based on the differences between RwO and RwS/RwP/RwT (see below).

Simulations were performed while introducing ablations that reduce the number of functioning neurons in certain buffers and associative memories. These ablations are designed to introduce dysfunctions with respect to the representations within specific buffers or with respect to the association of neural representations between two buffers. An ablation function is implemented in Nengo (Bekolay et al., 2014) and eliminates the neural activity of a specific percentage of randomly selected neurons within a chosen neural buffer or associative memory. With 0% ablation, the neural activity is unchanged, while with 100% ablation, no neurons in the buffer will ever have any activity.

Simulations are done using a model in which a specific percentage (from 0 to 100% in steps of 10%) of neurons are ablated within and between all three functional levels of the production pathway in the mental lexicon. Therefore, three *within-level dysfunctions* (a) within concept level dysfunction; c) within lemma level dysfunction; e) within phonological level dysfunction; see *concept_*prod; word_prod; phono_prod in Figure 1) and two *between-level dysfunctions* (b) between concept-to-lemma level dysfunction; d) between lemma-to-phonological level dysfunction; see the arrow between *concept_prod* and *word_prod*; *word_prod* and *phono_prod* in Figure 1) are defined. We have chosen these ablations due to the underlying literature on disorders within the mental lexicon that concern (i) storing semantic, lexical and phonological information, or (ii) accessing lexical-semantic or phonological form information for production (Best, 2005).

Buffers and associative buffers were also ablated individually to determine a direct connection between neural dysfunctions and behavioral outputs in the naming task with additional cues.

The data analysis is based on the same evaluation metric for each individual simulation. Each naming scenario is assessed using the correct/incorrect evaluation within the time window provided in the *phono_prod*-buffer (see Figures 2, 3). For an incorrect answer, zero points are awarded; for a correct answer, one point is awarded. An answer is rated as correct if activation of the correct S-pointer in the *phono_prod*-buffer is present over the appropriate time window (SPEAK). The activation needs to be above a threshold level of 0.2. Cues (RwS/RwP) and the control variable (RwT) are added later in the time course of individual simulation. The evaluation is also carried out for naming with cues or the control task via the correct/incorrect evaluation within the appertaining time window (SPEAK).

For the first experiment of the present work, the results of RwO_1-3 are compared with the norm data from the WWT. The norm data relates to the age category 5;6-6;5. A statistical comparison of the dysfunctions for the individual percentages was done using the Mann-Whitney *U*-test. For the second experiment, different aspects are concerned. In order to check whether there is a significant increase in naming performance from RwO_1-3 to RwS/RwP/RwT, the results are compared with the Wilcoxon signed-rank test for dependent, non-parametric samples. To determine whether the cues are influenced by the location of the dysfunction, a two-factor ANOVA is used with the factors *Type of Cue* (S/P) and *Location of Dysfunction* (a) -e)). To compare the different types of cues, the increase from RwO to RwS/RwP is compared with the increase of correctly named items from RwO to RwT and with each other using the Wilcoxon signed-rank test. All statistical analyzes are performed using SPSS Version 23 for Mac (SPSS Inc., Chicago, IL, United States). The individual level of significance is set to *p* = 0.05.

### Source Code

The source code for the simulations of the picture induced word naming (95 items) from the WWT is provided as additional material. The source code for the word naming task with semantic cues is labeled as WWT_semCue.ipynb. The source code for the word naming task with phonological cues is labeled as WWT_phonCue.ipynb. The source code for the control variable is labeled as WWT_noCue.ipynb. RwO and RwS/RwS/RwT were included consecutively in one simulation. Simulations were done using these ipython notebooks within the anaconda3 environment.

## Results

### Sample Simulation Results

To start, sample simulation results are presented to get insights into the detailed function of the model. Figure 2 shows a sample run that illustrates the RwO and the RwS. This sample run is used to explain how semantic cues work with active ablation in one buffer. The case of 50% ablation within the concept level buffer is chosen here. Simulations with and without cues are done consecutively in one simulation run. In RwO, the task is ‘PRODUCE_NOMEN’ (see *input_control* buffer). In the example in Figure 2 the target item “Kruecke” (“crutch”) is intended to be named. Additional visual input is “Beine” (“legs”), “Schuhe” (“shoes”), and “Mann” (“man”) according to the presented picture (see *visual_input* buffer). These additional visual inputs are not cues but are given in the target figure because the WWT uses photos which in contrast to simple line pictures may include non-important or even misleading details beyond the intended target concept. Cues are given in this example auditorily in RwS (see below). RwO refers to naming without semantic cues. The *action control* buffer ensures that the word is produced (S-pointer ‘SPEAK’) after processing the input (S-pointer ‘PROCESS_NOMEN’). The activation patterns associated with concept, lemma, and phonological form representations are displayed in the buffers *concept_prod*, *word_prod*, and *phono_prod*. In the *concept production* buffer, many other entries are also activated. This is due to semantic and associative connections within the mental lexicon. A cleanup memory between the *concept production* buffer and the *word production* buffer helps to select a single target item. In this case, the wrong item “Verbringen” (“to spend”) is selected. The activation pattern in the *phonological production* buffer, (see *phono_prod*) shows then that no item is passed, and therefore that no item is activated in RwO. Thus, cues are needed in order to produce the correct word in this simulation of the WWT.

The simulation extended and now labeled as RwS. The current task is ‘PRODUCE_NOMEN_CUE’ (see *input_control* buffer). The target word is still “crutch.” Thus, additional semantic cues occurring as auditory input for “crutch” are now presented for brief time periods as prescribed by the WWT: “HilfsmittelZumGehen” (“walking aid”); “StockMitGriffArmhalterung” (“Stick with handle arm holder”); “BrauchenWennNichtRichtigLaufen” (“need if not walking properly”; see *audio_in* buffer). Now, within the concept processing module, the presented cue phrases are routed to the *cue_in* and subsequently the *cue_out* buffers. During the routing from *cue_in* to *cue_out*, the cue phrases are translated into cue words - in this case, “aid,” “arm holder,” and “to walk” (see Table 3). These are passed from the *cue_out* buffer to the *concept_out* buffer. The *cue_out* buffer provides additional input to the *concept_out* buffer and thus can help to activate the intended item “crutch.” The *action control* buffer indicates that the output word is produced (S-pointer ‘SPEAK’) after processing the input (S-pointer ‘PROCESS_NOMEN_CUE’) as prescribed by the design of the WWT. The production of the word is displayed in the buffers *concept_prod*, *word_prod*, and *phono_prod* as was the case for RwO earlier. It can be seen that “aid” and “arm holder” show high activation in the *concept production* buffer and are the “winners” after the cleanup in the *word_prod* buffer, and are passed to the *phono_prod* buffer. These semantic cues are associated with “crutch” within the mental lexicon and cause the activation of the correct item over time (see *phono_prod* in Figure 2). Thus, this sample simulation indicates how semantic cues given as additional auditory input can facilitate the naming of words.

Figure 3 shows a sample run that illustrates how phonological cues can help in the case of 50% ablation in the concept production buffer. In this sample simulation, the item “Schaelen” (“to peel”) is the target word. Additional visual input is “Hand” (“hand”), “Kartoffel” (“potato”) and “Schaeler” (“peeler”) (see *visual_input* buffer). These additional visual inputs are not cues. Additional cues are given in this example auditorily in RwP (see below). RwO refers to naming without phonological cues. The presentation and the tasks are the same as in the example above, except for the *input_control* and *action_control* buffer. In this example, a verb is to be processed instead of a noun (e.g., PROCESS_VERB). In the case of the sample simulation shown here, the activation pattern in the *phonological production* buffer (see *phono_prod*) shows then that no item is activated. Is this case, the activation of “to peel” could not get the highest activation with RwO. Thus, cues are needed in order to produce the correct word in this simulation of the WWT.

Next, the simulation is further executed and labeled as RwP. The current task is now ‘PRODUCE_VERB_CUE’ (see *input_control* buffer). The target word is still “to peel.” The additional auditory phonological cue is the beginning of the target item (in this case “Sch” for the German word “schaelen”; /S/ in SAMPA transcription). This information is routed to the *cue_out* buffer and provides additional input to the *concept_out* buffer, and thus can help to activate the intended item “peel.” The production of the word is displayed in the buffers *concept_prod*, *word_prod*, and *phono_prod* as was the case for RwO earlier. The *concept production* buffer shows all activated semantic pointers. Next to “Sch,” “Schule,” (“school),” and “Schaelen” (“peel”), concepts like “Knirschen” (“crunch”), and “KnackenKnirschen” (“crack crunch”) are activated. “Crunch” and “crack” are associated with “break,” and “break” is associated with “chocolate” which starts in German with /sch/ (“Schokolade”). Phonological cues cause the model to produce the correct item in this later time period (see *phono_prod* in Figure 3). The effect of phonological cues seems to indicate that phonologically related entries are activated, which simultaneously activate their semantic counterparts at the *concept production* level. However, this can lead to the correct naming of the target object.

If the naming results in RwO or RwS/RwP/RwT are incorrect, the following errors are documented from all simulation results: No reaction, semantically related errors, unrelated errors, and individual syllables. No reaction or unrelated errors were the most common in our simulation. Phonological errors were not realized in any simulation.

### Experiment 1: Run Without Cues

The word naming task of the WWT 6-10 (95 items) was run three times using the normal model (0% ablation) and no cues. In the normal case, the model can on average correctly name 48 out of 95 items. The performance of the model is in the upper average range according to the norm data for the age category 5;6-6;5 (WWT, Glück, 2011).

**FIGURE 4:**
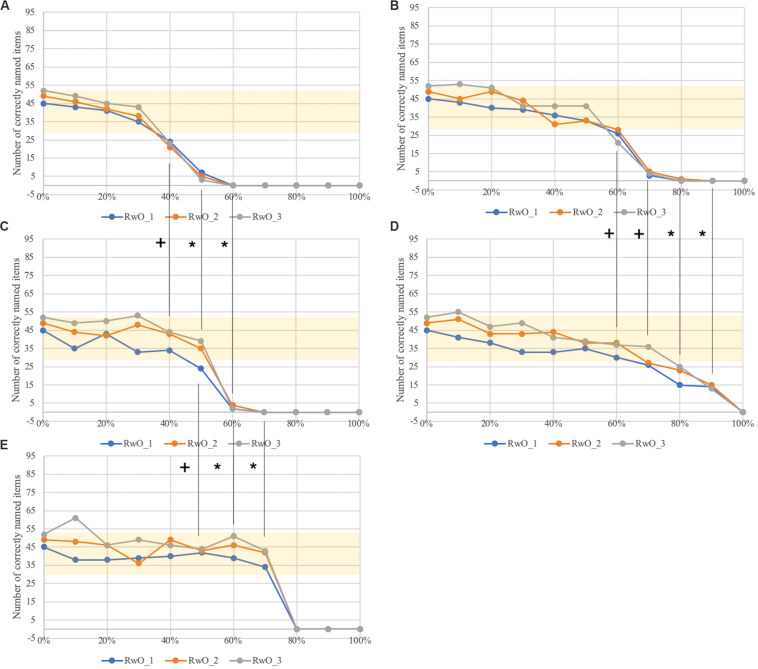
Number of correctly named items as a function of the percentage of ablated neurons in **(A)** concept buffer (concept_prod), **(C)** lemma buffer (word_prod), **(E)** lexeme buffer (phono_prod), and in the connection buffers between **(B)** the concept and the lemma buffer and between **(D)** the lemma and lexeme buffer. Statistical comparisons between the dysfunction conditions with the Mann-Whitney-*U*-test: + *p* = 0.05; ^∗^*p* < 0.05. The area marked in yellow shows the values of correctly named items within ± 1 standard deviation from the mean of the norm data for the age category 5;6-6;5 of the WWT.

The word naming task was further simulated without cues (RwO of WWT) for the five modeled pathological cases including different degrees of ablation (10% to 100%). The results are shown in Figure 4. It is observed that the model produces worse results in test performance for each of the five types of neural dysfunctions if the degree of dysfunction increases for a type of dysfunction. Further, different dysfunctions lead to different levels of test performance.

The lower levels of the mental lexicon corresponding to lemmas and phonological forms show better test performance than the highest level (i.e., the concept level) if the degree of dysfunction becomes stronger on each level. This is indicated by the statistical comparison of the within-level dysfunctions (a), c), e)). Between dysfunction a) and c) there is a statistical trend for 40% and 50% ablation and a significant difference for 60% ablation. The comparison of dysfunction c) and e) shows a statistical trend for 50% ablation and a significant difference for 60% and 70% ablation. The comparison of the between-level dysfunctions shows a statistical trend between the dysfunction b) and d) for the ablation values 60 and 70% and a significant difference for 80% and 90% ablation. Every comparison shows that a lower level of ablation corresponds to better performance.

### Experiment 2: Comparison Between Semantic and Phonological Cues

**FIGURE 5:**
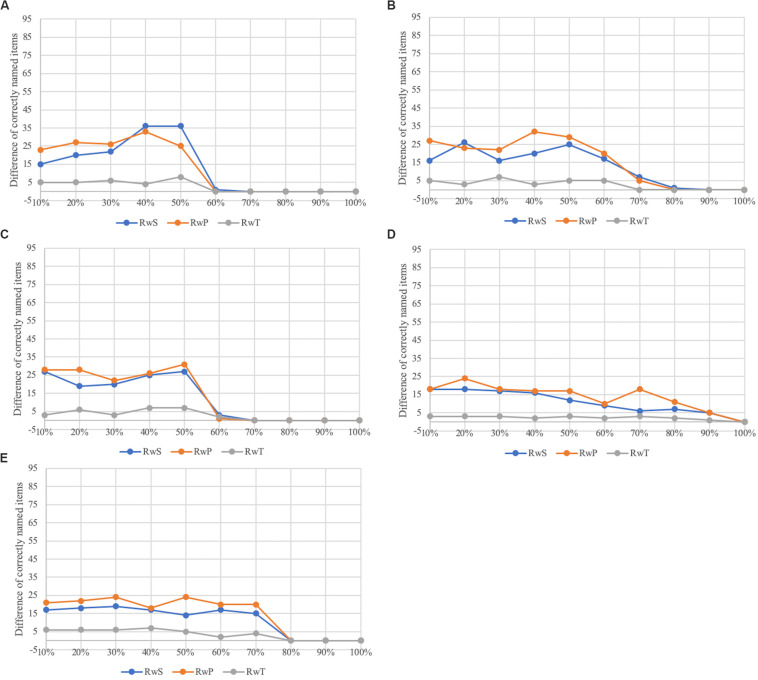
Difference values for the correctly named items between RwO and RwS (blue); RwO and RwP (orange) and RwO and RwT (gray) for the dysfunctions in different buffers and connections: **(A)** dysfunction at the concept level, **(B)** dysfunction between the concept and lemma levels, **(C)** dysfunction at the lemma level, **(D)** dysfunction between the lemma and lexeme levels, and **(E)** dysfunction at the lexeme level.

The word naming task was simulated without (RwO of WWT) and with semantic (RwS of WWT) and phonological cues (RwP of WWT) for the five modeled pathological cases including different degrees of ablation (10% to 100%). A set of runs (a run without cues and a run with cues for the same target item) was simulated as one unit. In other words, a run with cues for the same item always directly follows RwO for a given item (see Figures 2, 3). In order to investigate the effect of cues, a third naming run is carried out as a control (RwT). The results (differences between RwO and RwS/RwP/RwT) are shown in Figure 5 and Table 4.

**TABLE 4:** Mean number of correctly named items for the different runs (*RwS; RwP; RwT*) related to the different dysfunctions (a) to (e).

*Dys-func-tion*	*Number of correctly named items RwO_1 Mean ± SD*	*Number of correctly named items RwS Mean ± SD*	*Difference RwO vs. RwS Mean ± SD*	*RwO_1 vs. RwS p Value^1^for overall performance*	*Number of correctly named items RwO_2 Mean ± SD*	*Number of correctly named items RwP Mean ± SD*	*Difference RwO vs. RwP Mean ± SD*	*RwO_2 vs. RwP p value^1^for overall performance*	*Number of correctly named items RwO_3 Mean ± SD*	*Number of correctly named items RwT Mean ± SD*	*Difference RwO vs. RwT Mean ± SD*	*RwO_3 vs. RwT p value^1^for overall performance*	*Sem. vs. Phono. Cues (Difference Values) p value^1^for overall performance*	*Sem. vs. RwT s (Difference Values) p value^1^for overall performance*	*Phon. vs. RwT (Difference Values) p value^1^for overall performance*
a	15 ± 18.65	28 ± 29.71	13 ± 14.95	*p* = 0.03*	15.2 ± 19.66	28.7 ± 31.97	13.4 ± 14.35	*p* = 0.03*	16.3 ± 21.49	19.1 ± 23.61	2.8 ± 3.12	*p* = 0.02*	***p* = 0.75**	*p* = 0.03*	*p* = 0.04*
b	22 ± 18.86	34.8 ± 28.29	12.8 ± 10.08	*p* = 0.01*	23.6 ± 20.15	39.3 ± 32.48	15.8 ± 13.06	*p* = 0.02*	25.2 ± 22.51	27.6 ± 25.15	2.8 ± 2.66	***p* = 0.15**	***p* = 0.11**	*p* = 0.01*	*p* = 0.01*
c	17.1 ± 18.18	29.2 ± 29.96	12.1 ± 12.42	*p* = 0.03*	21.6 ± 22.18	35.2 ± 36.11	13.6 ± 14.3	*p* = 0.03*	23.7 ± 24.84	26.5 ± 27.26	2.8 ± 2.94	***p* = 0.14**	***p* = 0.14**	*p* = 0.03*	*p* = 0.04*
d	26.5 ± 12.92	37.3 ± 18.87	10.8 ± 6.34	*p* = 0.01*	32.2 ± 15.67	46 ± 22.22	13.8 ± 7.21	*p* = 0.01*	34.2 ± 17	36.4 ± 17.95	2.2 ± 1.03	***p* = 0.31**	*p* = 0.01*	*p* = 0.01*	*p* = 0.01*
e	27 ± 18.74	38.7 ± 26.81	11.7 ± 8.19	*p* = 0.02*	31 ± 21.7	45.9 ± 31.79	14.9 ± 10.44	*p* = 0.02*	34 ± 24	37.6 ± 26.48	3.6 ± 2.84	***p* = 0.71**	*p* = 0.01*	*p* = 0.01*	*p* = 0.01*

In addition, a statistical analysis to check the improvement in performance through cues (*RwO vs. RwS / RwP / RwT*), to check the effectiveness of the cues based on the difference values (*Sem. vs. Phono.)* and to check the effect using the comparison with the control variable (*RwS / RwP vs. RwT*). ^1^Wilcoxon signed-rank test, two- tailed. *p* = 0.05; * significant after correcting for multiple testing (a) within concept level dysfunction, (b) between concept-to-lemma level dysfunction, (c) within lemma level dysfunction, (d) between lemma-to-phonological level dysfunction, and (e) within phonological level dysfunction.

First, both cue types lead to significantly better performance for all defined dysfunctions a)-e) (see Table 4).

A two two-factor ANOVA is used with the factors *Type of Cue* (S/P) and *Location of dysfunction (a)-e))*. For all factors, the difference values between the with and without cue conditions were considered. *Type of Cue* resulted in one significant main effect (*F*(0,9) = 15,089, *p* = 0.004). The Location of Dysfunction was not significant (*p* > 0.05). The interaction between the two factors is not significant (*p* > 0.05). The pairwise comparison shows that semantic and phonological cues are significantly different in dysfunction d) and e) (for both *p* = 0.01).

The control variable leads only to significantly better performance for dysfunction a) (*p* = 0.02). However, semantic and phonological cues are significantly more effective at improving naming performance than the control task (Table 4).

### Performance for the Five Modeled Pathological Cases

To get insights, the individually defined dysfunctions are examined in a detailed analysis. Figures 4, 5 show the results for the simulated runs of within concept level dysfunction (a), between concept-to-lemma level dysfunction (b), within lemma level dysfunction (c), between lemma-to-phonological level dysfunction (d), and within phonological level dysfunction (e). Figure 4 shows of the results of the word naming task simulated without cues (RwO of WWT), while Figure 5 shows the difference between naming without and with semantic and phonological cues and the control for the five modeled pathological cases with different degrees of ablation (0% to 100%). The presentation of the results is divided into within-level dysfunctions and between-level dysfunctions.

#### Within-Level Dysfunctions:

##### Within conceptual level dysfunction

Figure 4A shows that with ablation in the concept buffer up to 30%, there is only a slight drop in correctly named items. All values are in the normal range after evaluation using the WWT criteria (age category 5;6-6;5). With between 30 and 60% ablation, there is a sharp drop toward zero correctly named items at 60% ablation. Across all ablation values and all simulation runs (RwO_1-3), an average of 15.5 (with a standard deviation (SD) of 19.28) items can be correctly named.

Figure 5A shows an increase in difference values for up to 40% ablation with semantic cues (blue line). This is followed by a sharp drop to a difference of zero at 60% ablation. Semantic cues lead to a maximum difference between the correctly named items of 36 items at 40% and 50% ablation, such that with semantic cues 36 more items can be named than without cues. On average, there is an improvement of 13 (SD 14.95) items with dysfunctions at the concept level, which can be named more with than without semantic cues (see Table 4).

Phonological cues (orange line) lead to an increase in correctly produced items between 10 and 40% ablation to 33. From an ablation of 40%, the difference between the correctly named items in RwO and RwP decreases to zero at 60% ablation. On average, 13.4 (SD 14.35) additional items can be named using phonological cues (see Table 4).

The control variable shows little improvement by allowing more time to produce the target item. On average, 2.8 more (SD 3.12) items can be named.

The comparison between semantic and phonological cues shows that phonological cues up to 30% ablation lead to a better difference rate. In contrast, semantic cues are superior in the 40% - 50% range. The statistical comparison shows no significant difference between the two types of cues when there is a dysfunction within the concept level.

##### Within lemma level dysfunction

In the case of up to 50% ablation in the lemma buffer (Figure 4C), there is only a slight drop and in some cases an increase in the number of correctly named items. All values up to 50% ablation (except one value from RwO_1), are in the normal range after evaluating with the WWT criteria (age category 5;6-6;5). With between 50 and 70% ablation, there is a sharp drop toward zero correctly named items at 70% ablation. Across all ablation values and all simulation runs, an average of 20.8 (with a standard deviation of 21.32) items can be correctly named.

Figure 5C shows an increase in the difference values for semantic and phonological cues up to 50% ablation. Then there is a sharp drop to difference values of zero and one at 60% ablation. Semantic cues (blue line) lead to a maximum difference of 27 correctly named items at 50% ablation. On average, there is an increase of 12.1 (SD 12.42) named items for this lemma level dysfunction using semantic cues (see Table 4).

Phonological cues (orange line) lead to an increase in correctly produced items at 50% ablation, up to a maximum improvement of 31. On average, an additional 13.6 (SD 14.3) items can be named using phonological cues (see Table 4).

The control variable shows little improvement by ensuring more time to produce the target item. On average, 2.8 (SD 2.94) more items can be named.

The comparison between semantic and phonological cues shows that phonological cues with between 10 and 50% ablation lead to a better difference rate. The statistical comparison shows no significant difference between the two types of cues when there is a dysfunction within the lemma level.

##### Within phonological level dysfunction

In the case of ablation in the lexeme buffer (Figure 4E), up to about 70% ablation, there is only a slight drop and in some cases an increase in the number of correctly named items. All values of ablation up to 70% yield results in the normal range after the evaluation with the WWT criteria (age category 5; 6-6; 5). Between 70 and 80% ablation, there is a sharp drop toward zero correctly named items at 80%. Across all ablation values and all simulation runs, an average of 30.67 (with a standard deviation of 21.03) items can be correctly named.

Figure 5E shows a stable course for semantic and phonological cues with between 10 and 70% ablation. We observe positive difference values between 14 and 19 for the semantic cues (blue line) and between 18 to 24 for phonological cues (orange line). From 70% ablation, there is a drop to a difference value of zero at 80% ablation. Semantic cues lead to an average improvement of 11.7 (SD 8.19) additional named items (see Table 4). Phonological cues lead to an average improvement of 14.9 (SD 10.44) additional named items (see Table 4).

The control variable again shows little improvement by ensuring more time to produce the target item. On average, 3.6 (SD 2.84) more items can be named.

The comparison between semantic and phonological cues shows that phonological cues lead to a better difference rate across all ablation values. The statistical comparison shows that there is a significant difference between the two types of cues (see Table 4).

#### Between-Level Dysfunctions

##### Between conceptual and lemma level dysfunction

Figure 4B shows a slight drop off up to 50% ablation. Between 50 and 70% ablation, there is a greater drop in the number of correctly named items. Between 70 and 90% ablation, there is a further drop which results in zero correctly named items at 90% ablation. Results with 60% ablation are below the normal range as per the WWT criteria (age category 5;6-6;5). Across all ablation values and all simulation runs, an average of 22 (SD 18.86) items can be correctly named.

Figure 5B shows a moderate increase in the difference values for semantic cues (blue line) from 10 to 20% ablation and from 30 to 50% ablation. Then there is a slight decrease to a difference of zero at 90% ablation. Semantic cues lead to a maximum difference 26 correctly named items at 20% ablation. On average, there is an improvement of 12.8 (SD 10.08) correctly named items for this dysfunction, with the use of semantic cues increasing correct naming (see Table 4).

Phonological cues (orange line) lead to an increase of 32 correctly produced items between 10 and 40% ablation. From 40% ablation, the difference between the correctly named items decreases to zero at 80% ablation. On average, 15.8 (SD13.06) additional items can be named using phonological cues (see Table 4).

The control variable shows little improvement by ensuring more time to produce the target item. On average, 2.8 (SD 2.66) more items can be named.

The comparison between semantic and phonological cues shows that phonological cues at 10% ablation and between 30 and 60% ablation lead to a better difference rate. At 20%, 70%, and 80% ablation, semantic cues are slightly superior. The statistical comparison shows that there is no significant difference between the two types of cues when there is a dysfunction between the concept and lemma level.

##### Between lemma and phonological level dysfunction

Figure 4D shows a shallow drop to zero correctly named items at 100% ablation. Results from 70% ablation are below the normal range after evaluating with the WWT criteria (age category 5;6-6;5). Overall all ablation values, an average of 30.97 (SD 15.15) items can be correctly named.

Figure 5D shows a slight decrease in the difference values for semantic cues between 10 and 100% ablation (blue line). Semantic cues lead to a maximum difference of 18 correctly named items at 10% and 20% ablation. On average there is an improvement of 10.8 (SD 6.34) correctly named items for this dysfunction with semantic cues (see Table 4).

The difference values for the phonological cues (orange line) show a slight increase up to 20% ablation, followed by a slight drop toward 60% ablation. At 70% ablation, there is a further increase with a difference value of 18. There is a gentle decrease in the difference value to zero at 100% ablation. Phonological cues lead to a maximum increase of 24 correctly produced items at 20% ablation. On average, 13.8 (SD 7.21) additional items can be named using phonological cues (see Table 4).

The control variable shows little improvement by ensuring more time to produce the target item. On average, 2.2 (SD 1.03) more items can be named.

The comparison between semantic and phonological cues shows that phonological cues lead to a better difference rate across all ablation values. The statistical comparison shows that there is a significant difference between the two types of cues (see Table 4).

## Discussion

The aims of this study were to analyze the behavioral effects of different modeled dysfunctions within and between different levels the mental lexicon, and to measure the effects of semantic and phonological cues. The simulation method used allows us to associate clearly defined neural deficits with behavioral deficits that result directly from the simulations.

The model incorporates the mental lexicon as part of long-term and working memory. A knowledge repository with three levels (concept, lemma, and phonological) is implemented as long-term memory. Small-capacity working memories are able to represent the current cognitive state by storing a small amount of information over a brief period of time. The concept and phonological form surface networks and the lemma network can be accessed in the mental lexicon and activated in the respective production or perception pathway neural buffers, as well as in concept buffers within the cognitive processing module. The neural realization of the speech processing model is based on a model of spiking neurons (the leaky integrate-and-fire model) incorporated into neuron ensembles, which are then organized into neuron buffers. The neuron buffers represent high-dimensional cognitive, motor, and sensory states. Associative memories represent mappings between buffers. With this modeling approach, speech processing within a buffer and between two buffers can be clearly separated. Furthermore, buffers and associative memories can be ablated to different degrees and the resulting neural deficits can be precisely defined and localized. Therefore, neuronal deficits in different parts (different levels or connections) of our model can be defined in order to be able to investigate the influence of these deficits on behavior in different simulated speaking tasks.

Ablation is introduced in the present model as a basic approach for modeling neural deficits (microscopic dysfunctions) that occur within and between all three functional levels of the production pathway in the mental lexicon. This accounts for the distinction between semantic and phonological level, and also the distinction between storage and access disorders. The ablation function silences a specific percentage of randomly selected neurons within a chosen neural buffer.

In this study, two experiments were performed using increasing levels of ablation within and between different neural buffers. One experiment concerns the naming task without (RwO) cues and the other experiment concerns naming with semantic (RwS) and phonological cues (RwP). In order to investigate the effect of cues, a third naming run is carried out as a control variable (RwT). The results are compared and discussed (see below) with respect to the norm data of WWT 6-10 and with studies in the field of SLI and aphasia. Even if the test and the test results relate to children’s speech disorders, studies on aphasia are able to explain the simulation results, in particular the effectiveness of the cues (Best, 2005; Friedmann and Novogrodsky, 2008; Novogrodsky et al., 2010).

The first experiment in our work serves to check the model with regard to comparability with the norm data of the used test methods. The model is instructed to name 95 words based on specific visual and auditory inputs (RwO of WWT 6-10, Glück, 2011). Since behavioral data are available for these test methods, we can test whether the neural model behaves “normally” or “pathologically.” This enables us to evaluate the quality and realism of the neural model (Stille et al., 2019). The results of the present simulations for WWT 6-10 show that the model can on average correctly name 48 of 95 items in the normal case (0% ablation). The results are in the normal range for the age category 5;6 (5 years and 6 months) to 6;5 (6 years and 5 months). In summary, the results show that the model is able to generate behavioral data in a normal range and can thus simulate test behavior in humans without a lexical disorder (Kröger et al., 2016; Stille et al., 2019).

Further behavioral data in the context of differently defined neuronal deficits were simulated. For this purpose, specific neuronal deficits were introduced into the model, which lead to specific deviations in the behavior produced by the simulations. Five different neural dysfunctions have been defined in relation to the production side of the neural model. Three of these dysfunctions are *within-level dysfunctions* (concept, lemma, and phonological level) and two are *between-level dysfunctions* (concept-to-lemma and lemma-to-phonological).

The between-level dysfunctions are more robust compared to the within-level dysfunctions. Higher ablation values between 80 and 90% still result in correctly named. This type of dysfunction can be understood in relation to the retrieval hypothesis. Here, the mental lexicon is comparable to that of a normally developed child. However, lexical retrieval is less efficient (Fried-Oken, 1987; Newman and German, 2002). Results from the model show that intact levels of the mental lexicon can compensate for between-level dysfunctions with high ablation values. The entries are activated more effectively if the storage of semantic, lexical and phonological knowledge is intact.

In addition to these results, the higher levels of the model (i.e., the lemma and concept levels) appear to be more susceptible to the severity of the disorder than the lower levels. The mean number of correctly named items over three runs are higher for the lower levels (lemma and phonological) than for the concept level. This result is underpinned by the statistical trend and significant difference between a dysfunction at the concept and lemma level (between 40 and 60% ablation) and between the lemma and lexeme level (between 50 and 80% ablation). The two between-level dysfunctions also differ significantly between 60 and 90% ablation. In all comparisons, the lower levels perform better. The comparison with the norm data of WWT 6-10 supports this result. For example, performance at up to 30% ablation in concept level is in the normal range, while performance at up to 70% ablation in the lexeme level is within this range. This result shows that with a serial process of speech production (Levelt et al., 1999) as in the present model, dysfunctions in the higher levels significantly impair processing in a word naming tasks. Dysfunctions at lower levels seem to allow for the activation of compensation patterns through intact information drawn from the higher levels. Natural data show that children with word-finding disorders make fewer phonological mistakes than semantic mistakes, but significantly more phonological mistakes in comparison to the control groups (age control group; control group that has the same grammatical skills; same naming achievement) (Dockrell et al., 2001; McGregor and Appel, 2002). Semantic errors can arise from deficits in the semantic representation of the word and from impaired access to its phonological representation (Caramazza and Hillis, 1990; McGregor, 1997; Howard and Gatehouse, 2006; Biran et al., 2018). On the other hand, phonological errors can only arise from a disturbance at the lexeme level or in access to it (German, 2002). It can be concluded that if children with speech development disorders and word-finding disorders show fewer phonological errors, fewer disorders of the lexeme level occur or, as per the results of the model, disorders at the lexeme level can be compensated for at increasing levels of severity.

Overall, the model’s simulated behavioral results show that ablations of different buffers have different behavioral effects, and that test performance weakens with increasing difficulty. This highlights the idea that different functional modules of our speech processing model are reacting with different sensitivity to specific dysfunctions. It also highlights that different areas of our model take on different roles in the word production process (Levelt et al., 1999). Since the presented model is biologically inspired and based on known facts about the physiology of speech processing, the model buffers are not assigned to specific regions but are defined functionally. Hypothetical assignments of the localization of the mental lexicon and the speech processing in the brain are examined in several imaging studies (for an overview see Indefrey and Levelt, 2000, 2004; Indefrey, 2011).

The results of the second experiment show that semantic and phonological cues improve naming significantly in the case of all defined neural disorders. Semantic and phonological cues activate the reconstruction of information that was not accessible directly (in RwO). This is confirmed by the significant differences observed in comparison to the control variable. The cause of these results might be that semantic or phonological cues activate lexical associations and thus lead to the activation of bundles of associated items at different lexical levels. This reduces the selection of possible lexical entries and thus facilitates word production and increases the probability of a correct naming as proposed in the literature (Nickels and Best, 1996; Brackenbury and Pye, 2005; Gershkoff-Stowe and Hahn, 2007; Velez and Schwartz, 2010; phonological cues: German, 2002; spreading activation theory: Collins and Loftus, 1975). Concepts that are semantically or phonologically related are neutrally associated (neutrally connected). When a cue related to a target word is given, the target word gets additional neural activation.

With respect to the difference between storage and access disorders, these results show that cues are effective in the case of an accessing disorder (between-level dysfunction) and in the case of dysfunction that interferes with ablated neuronal activity within a level (within-level dysfunction). It can be assumed that for within-level dysfunction, it is not the storage itself that is disturbed, but the association between the surface and deep layer and therefore the organization of the corresponding buffer and thus also lexical access. Representations might be primarily inaccessible in all defined dysfunctions (Gershkoff-Stowe and Smith, 1997; Gershkoff-Stowe, 2002; Jefferies and Lambon Ralph, 2005, 2006; Abel et al., 2007).

A further aim of our study is to compare the effectiveness of semantic and phonological cues in the context of different neural dysfunctions. It has been hypothesized that semantic cues are more effective for patients with semantic disorders, while phonological cues are more effective for patients with phonological disorders (Hickin et al., 2002; Van Hees et al., 2013). Our results indicate that semantic cues have the greatest effect in cases that involve concept level neural dysfunction. The differences between naming with and without semantic cues for the other neural dysfunctions are lower but more robust at progressively higher levels of the dysfunction, especially in the case of dysfunctions between the concept-to-lemma and lemma-to-phonological levels. The most long-lasting improvement (due to the ablation values) is in the case of between lemma-to-phonological level dysfunction (still 5 improvements in case of 90% of ablation). This shows that semantic cues can improve naming with between-level dysfunction if the storage of semantic, lexical and phonological knowledge is functioning properly even if the ablation values are very high (i.e., 70 or 90%). In the case of within concept and lemma level dysfunctions and 60% and 70% ablation, semantic cues no longer improve naming performance. This means that the number of intact neural connections within the buffer is important for allowing semantic cues to work and have a facilitating effect on naming. However, these differences are not significant. In general, semantic cues have the greatest effect on higher levels and lesser effects on lower levels, though they still produce the facilitation effect.

Phonological cues produce long-lasting improvements over multiple ablation values in the case of between lemma-to-phonological level dysfunction (still 5 improvements with 90% ablation). A great improvement was found with phonological cues in the case of between concept-to-lemma level dysfunction and within phonological level dysfunction. Moreover, in the case of phonological cues, performance for between-level dysfunctions remains more robust at progressively higher levels of ablation. This shows that cues can improve naming with between-level dysfunction if the storage of semantic, lexical and phonological knowledge is functioning properly even if the ablation values are very high (i.e., 70 to 90%). In the case of within concept and lemma level dysfunctions and 60 or 70% ablation, phonological cues no longer improve naming performance. In general, phonological cues have the greatest effect on lower levels in comparison to higher levels. Nevertheless, both semantic and phonological cues have an effect on both lower and higher levels. This shows that the associations within and between different levels of the mental lexicon are activated by each process of word access.

The direct comparison of the difference between naming with and without phonological cues and the difference between naming with and without semantic cues indicate that on average phonological cues are more effective than semantic cues at facilitating naming in all defined lexical dysfunctions. The only cases in which semantic cues are more effective than phonological cues are if a neural deficit is located strictly in the concept storage area and if higher levels of ablation are present in the mental lexicon (40% and 50% ablation). The statistical comparison shows a main effect for the type of cue. The pairwise comparison shows that phonological cues lead to a significantly greater difference between naming with and without cues when the phonological level is ablated. From this, it can be concluded not only that phonological cues activate phonologically similar items, but that phonological cues also support processing at higher levels when accessing the mental lexicon. This includes processing that takes place within the lemma level and between the concept-to-lemma level and the lemma-to-phonological level. These findings can be explained by simulation results (Figure 3). Phonological cues already activate phonologically related items and their semantic relations in the *conceptual production* buffer (see Figure 3 and Tables 1, 2). Here the semantic system of phonological neighbors is activated. This shows that lexical access is highly interactive in nature (Wambaugh et al., 2001) and in our computer-simulated model. Furthermore, this observation underpins the idea that different layers within the mental lexicon are not acting totally independently from each other. Speech processing and lexical access are realized in complex interacting networks and not by different independent networks (Stella et al., 2018). This conclusion is also underpinned by the fact that in the literature, a benefit of phonological cues can be found in patients with aphasia and children with SLI (aphasia: Li and Williams, 1991; Stimley and Noll, 1991; McGregor, 1997; German, 2002; Wambaugh, 2003; Meteyard and Bose, 2018) because these language disorders are related to higher-level processing deficits and not to phonological level dysfunctions. This is mirrored in our model simulations as well. It can be seen from our simulations that phonological cues facilitate both the categorization of the target word and the phonological output, making it more useful for image naming than semantic cues (see also the experimental results reported by Meteyard and Bose, 2018).

## Conclusion

The goal of our research was to discover the underlying neural functional deficits which cause specific behavioral deficits as quantified in a naming task without and with semantic and phonological cues. In general: naming tasks with or without helping cues provide a valuable simulation scenario that can help investigate the relationship between neural dysfunctions and corresponding behavioral deficits or language disorders. It is only with precise modeling and simulation that neural dysfunctions can be clearly defined. The association of specific neural deficits with behavioral deficits in the form of lexical dysfunctions cannot be proven with real patients, because in this case the disorders related to the access of the mental lexicon cannot be defined as clearly as it is possible to do by using a model.

Moreover, cueing and priming offer an effective way to examine word retrieval in the mental lexicon (Lucas, 2000). From our simulation results, we can conclude that phonological cues are more effective than semantic cues as is hypothesized in other studies (Meteyard and Bose, 2018). Phonological cues seem not only to activate phonologically similar items; they also support higher-level processing during access of the mental lexicon. These processes occur within the lemma level as well as in connections between the concept and lemma levels, and between the lemma and phonological levels.

## Data Availability Statement

The datasets generated for this study are available on request to the corresponding author.

## Author Contributions

BK and CS contributed to planning the study. BK, TB, PB, and CS contributed to software coding. BK and CS conducted the simulation experiments. CS wrote the manuscript. All authors contributed to correcting the manuscript.

## Conflict of Interest

PB and TB are employed by Applied Brain Research, Inc., Waterloo, ON, Canada. The remaining authors declare that the research was conducted in the absence of any commercial or financial relationships that could be construed as a potential conflict of interest.

## References

[B1] Abel S. Willmes K. Huber W. (2007). Model-oriented naming therapy: testing predictions of a connectionist model. *Aphasiology* 21 411–447. 10.1080/02687030701192687

[B2] Alt M. Plante E. (2006). Factors that influence lexical and semantic fast mapping of young children with specific language impairment. *J. Speech Lang. Hear. Res.* 49 941–954. 10.1044/1092-4388(2006/068) 17077207

[B3] Archibald L. M. Gathercole S. E. (2007). The complexities of complex memory span: storage and processing deficits in specific language impairment. *J. Mem. Lang.* 57 177–194. 10.1016/j.jml.2006.11.004

[B4] Baddeley A. (2010). Working memory. *Curr. Biol.* 20 R136–R140. 20178752 10.1016/j.cub.2009.12.014

[B5] Bekolay T. Bergstra J. Hunsberger E. DeWolf T. Stewart T. C. Rasmussen D. (2014). Nengo: a python tool for building large-scale functional brain models. *Front. Neuroinform.* 7:48. 10.3389/fninf.2013.00048 24431999 PMC3880998

[B6] Best W. (2005). Investigation of a new intervention for children with word-finding problems. *Int. J. Lang. Commun. Disord.* 40 279–318. 10.1080/13682820410001734154 16195190

[B7] Biran M. Novogrodsky R. Harel-Nov E. Gil M. Mimouni-Bloch A. (2018). What we can learn from naming errors of children with language impairment at preschool age. *Clin. Linguist. Phon.* 32 298–315. 10.1080/02699206.2017.1365096 28853966

[B8] Blouw P. Solodkin E. Thagard P. Eliasmith C. (2016). Concepts as semantic pointers: a framework and computational model. *Cogn. Sci.* 40 1128–1162. 10.1111/cogs.12265 26235459

[B9] Brackenbury T. Pye C. (2005). Semantic deficits in children with language impairments: issues for clinical assessment. *Lang. Speech Hear. Serv. Sch.* 36 5–16. 10.1044/0161-1461(2005/002) 15801504

[B10] Bragard A. Schelstraete M. A. (2007). Word-finding difficulties in French-speaking children with SLI: a case STUDY. *Clin. Linguist. Phon.* 21 927–934. 10.1080/02699200701615211 17972189

[B11] Brendel B. Erb M. Riecker A. Grodd W. Ackermann H. Ziegler W. (2011). Do we have a “mental syllabary” in the brain? An fMRI study. *Mot. Control* 15 34–51. 10.1123/mcj.15.1.34 21339513

[B12] Butterworth B. (1989). “Lexical access in speech production,” in *Lexical Representation and Process*, ed. Marslen-Wilson W., (Cambridge, MA: MIT Press), 108–135.

[B13] Caramazza A. (1997). How many levels of processing are there in lexical access? *Cogn. Neuropsychol.* 14 177–208. 10.1080/026432997381664

[B14] Caramazza A. Hillis A. E. (1990). Levels of representation, co-ordinate frames, and unilateral neglect. *Cogn. Neuropsychol.* 7 391–445. 10.1080/02643299008253450

[B15] Cholin J. (2008). The mental syllabary in speech production: an integration of different approaches and domains. *Aphasiology* 22 1127–1141. 10.1080/02687030701820352

[B16] Collins A. M. Loftus E. F. (1975). A spreading-activation theory of semantic processing. *Psychol. Rev.* 82 407–428. 10.1037/0033-295x.82.6.407

[B17] Crawford E. Gingerich M. Eliasmith C. (2016). Biologically plausible, human-scale knowledge representation. *Cogn. Sci.* 40 782–821. 10.1111/cogs.12261 26173464

[B18] Dell G. S. (1986). A spreading-activation theory of retrieval in sentence production. *Psychol. Rev.* 93:283 10.1037/0033-295x.93.3.283 3749399

[B19] Dell G. S. O’Seaghdha P. G. (1992). Stages of lexical access in language production. *Cognition* 42 287–314. 10.1016/0010-0277(92)90046-k 1582160

[B20] Dell G. S. Schwartz M. F. Martin N. Saffran E. M. Gagnon D. A. (1997). Lexical access in aphasic and nonaphasic speakers. *Psychol. Rev.* 104:801. 10.1037/0033-295x.104.4.801 9337631

[B21] Dockrell J. E. Messer D. George R. (2001). Patterns of naming objects and actions in children with word finding difficulties. *Lang. Cogn. Process.* 16 261–286. 10.1080/01690960042000030

[B22] Eliasmith C. (2012). A large-scale model of the functioning brain. *Science* 338 1420–1420. 10.1126/science.1225266 23197532

[B23] Eliasmith C. (2013). *How to Build a Brain: A Neural Architecture for Biological Cognition.* New York, NY: Oxford University Press.

[B24] Eliasmith C. Anderson C. H. (2003). *Neural Engineering: Computation, Representation, and Dynamics in Neurobiological Systems.* Cambridge, MA: MIT Press.

[B25] Eliasmith C. Gosmann J. Choo X. (2016). BioSpaun: a large-scale behaving brain model with complex neurons. *arXiv* [Preprint]. Available at: https://arxiv.org/abs/1602.05220 (accessed Jan 6, 2020).

[B26] Eliasmith C. Stewart T. C. Choo X. Bekolay T. DeWolf T. Tang Y. (2012). A large-scale model of the functioning brain. *Science* 338 1202–1205. 10.1126/science.1225266 23197532

[B27] Elman J. L. (2004). An alternative view of the mental lexicon. *Trends Cogn. Sci.* 8 301–306. 10.1016/j.tics.2004.05.003 15242689

[B28] Foygel D. Dell G. S. (2000). Models of impaired lexical access in speech production. *J. Mem. Lang.* 43 182–216. 10.1006/jmla.2000.2716 26555757

[B29] Friedmann N. Novogrodsky R. (2008). “Subtypes of SLI: SySLI, PhoSLI, LeSLI, and PraSLI,” in *Language Acquisition And Development*, eds Gavarró A. João Freitas M., (Cambridge: Cambridge Scholars Press/CSP), 205–217.

[B30] Fried-Oken M. (1987). Qualitative examination of children’s naming skills through test adaptations. *Lang. Speech Hear. Serv. Sch.* 18 206–216. 10.1044/0161-1461.1803.206

[B31] Garrett M. (1980). “Levels of processing in sentence production,” in *Language Production*, ed. Butterworth B., (London: Academic Press).

[B32] German D. J. (2000). *Test of Word Finding*, 2nd Edn, Austin, TX: PRO-ED.

[B33] German D. J. (2002). A phonologically based strategy to improve word-finding abilities in children. *Commun. Disord. Q.* 23 177–190. 10.1177/15257401020230040301

[B34] Gershkoff-Stowe L. (2002). Object naming, vocabulary growth, and the development of word retrieval abilities. *J. Mem. Lang.* 46 665–687. 10.1006/jmla.2001.2830

[B35] Gershkoff-Stowe L. Hahn E. R. (2007). Fast mapping skills in the developing lexicon. *J. Speech Lang. Hear. Res.* 50 682–697. 10.1044/1092-4388(2007/048) 17538109

[B36] Gershkoff-Stowe L. Smith L. B. (1997). A curvilinear trend in naming errors as a function of early vocabulary growth. *Cogn. Psychol.* 34 37–71. 10.1006/cogp.1997.0664 9325009

[B37] Glück C. W. (2011). *Wortschatz- und Wortfindungstest Für 6-10-Jährige.* Amsterdam: Elsevier.

[B38] Gray S. (2005). Word learning by preschoolers with specific language impairment: Effect of phonological or semantic cues. *J. Speech Lang. Hear. Res.* 48 1452–1467. 10.1044/1092-4388(2005/101) 16478383

[B39] Hickin J. Best W. Herbert R. Howard D. Osborne F. (2002). Phonological therapy for word-finding difficulties: a re-evaluation. *Aphasiology* 16 981–999. 10.1080/02687030244000509

[B40] Howard D. Gatehouse C. (2006). Distinguishing semantic and lexical word retrieval deficits in people with aphasia. *Aphasiology* 20 921–950. 10.1080/02687030600782679

[B41] Indefrey P. (2011). The spatial and temporal signatures of word production components: a critical update. *Front. Psychol.* 2:255 10.3389/fninf.2013.000255 PMC3191502 22016740

[B42] Indefrey P. Levelt W. J. (2000). “The neural correlates of language production,” in *The New Cognitive Neurosciences*, 2nd Edn, ed. Gazzaniga M. S., (Cambridge, MA: MIT press), 845–865.

[B43] Indefrey P. Levelt W. J. (2004). The spatial and temporal signatures of word production components. *Cognition* 92 101–144. 10.1016/j.cognition.2002.06.001 15037128

[B44] Jacquemot C. Scott S. K. (2006). What is the relationship between phonological short-term memory and speech processing? *Trends Cogn. Sci.* 10 480–486. 10.1016/j.tics.2006.09.002 16997610

[B45] Jefferies E. Lambon Ralph M. A. (2005). Non-verbal semantic impairment in stroke aphasia: a comparison with semantic dementia. *Brain Lang.* 95:244ff. 10.1093/brain/awl153 16815878

[B46] Jefferies E. Lambon Ralph M. A. (2006). Semantic impairment in stroke aphasia versus semantic dementia: a case-series comparison. *Brain* 129 2132–2147. 10.1093/brain/awl153 16815878

[B47] Kail R. Leonard L. B. (1986). *Word-Finding Abilities In Language-Impaired Children (ASHA Monograph, No. 25).* Rockville, MD: American Speech-Language-Hearing Association. 3730040

[B48] Kröger B. J. Bafna T. Cao M. (2019). Emergence of an action repository as part of a biologically inspired model of speech processing: the role of somatosensory information in learning phonetic-phonological sound features. *Front. Psychol.* 10:1462. 10.3389/fpsyg.2019.01462 31354560 PMC6635888

[B49] Kröger B. J. Cao M. (2015). The emergence of phonetic–phonological features in a biologically inspired model of speech processing. *J. Phon.* 53 88–100. 10.1016/j.wocn.2015.09.006 PMC6635888 31354560

[B50] Kröger B. J. Crawford E. Bekolay T. Eliasmith C. (2016). Modeling interactions between speech production and perception: speech error detection at semantic and phonological levels and the inner speech loop. *Front. Comput. Neurosci.* 10:51. 10.3389/fninf.2013.00051 27303287 PMC4885855

[B51] Laine M. Martin N. (2006). *Anomia: Theoretical and Clinical Aspects.* Hove: Psychology Press.

[B52] Levelt W. J. M. (1989). *Speaking: From Intention To Articulation.* Cambridge, MA: MIT Press.

[B53] Levelt W. J. M. Roelofs A. Meyer A. S. (1999). A theory of lexical access in speech production. *Behav. Brain Sci.* 22 1–75. 11301520 10.1017/s0140525x99001776

[B54] Li E. C. Williams S. E. (1991). An investigation of naming errors following semantic and phonemic cueing. *Neuropsychologia* 29 1083–1093. 10.1016/0028-3932(91)90078-m 1723180

[B55] Lorenz A. Ziegler W. (2009). Semantic vs. word-form specific techniques in anomia treatment: a multiple single-case study. *J. Neurolinguistics* 22 515–537. 10.1016/j.jneuroling.2009.05.003

[B56] Lucas M. (2000). Semantic priming without association: a meta-analytic review. *Psychon. Bull. Rev.* 7 618–630. 10.3758/bf03212999 11206202

[B57] Martin N. Laine M. (2000). Effects of contextual priming on impaired word retrieval. *Aphasiology* 14 53–70. 10.1080/026870300401595

[B58] McGregor K. K. (1997). The nature of word-finding errors of preschoolers with and without word-finding deficits. *J. Speech Lang. Hear. Res.* 40 1232–1244. 10.1044/jslhr.4006.1232 9430745

[B59] McGregor K. K. Appel A. (2002). On the relation between mental representation and naming in a child with specific language impairment. *Clin. Linguist. Phon.* 16 1–20. 10.1080/02699200110085034 11913029

[B60] McGregor K. K. Waxman S. R. (1998). Object naming at multiple hierarchical levels: a comparison of preschoolers with and without word-finding deficits. *J. Child Lang.* 25 419–430. 10.1017/s030500099800347x 9770914

[B61] McGregor K. K. Windsor J. (1996). Effects of priming on the naming accuracy of preschoolers with word-finding deficits. *J. Speech Lang. Hear. Res.* 39 1048–1058. 10.1044/jshr.3905.1048 8898257

[B62] Meteyard L. Bose A. (2018). What does a cue do? comparing phonological and semantic cues for picture naming in aphasia. *J. Speech Lang. Hear. Res.* 61 658–674. 10.1044/2017_jslhr-l-17-0214 29486495

[B63] Newman R. S. German D. J. (2002). Effects of lexical factors on lexical access among typical language-learning children and children with word-finding difficulties. *Lang. Speech* 45 285–317. 10.1177/00238309020450030401 12693688

[B64] Nickels L. Best W. (1996). Therapy for naming disorders (Part I): Principles, puzzles and progress. *Aphasiology* 10 21–47. 10.1080/02687039608248397

[B65] Novogrodsky R. Kreiser V. Friedmann N. (2010). The heterogeneity within the lexical deficit in SLI. *Paper Presented at the 31st annual Symposium on Research in Child Language Disorders*, Madison.

[B66] SAMPA (2005). *SAMPA–Computer Readable Phonetic Alphabet (Last Revised 2005).* Available at: https://www.phon.ucl.ac.uk/home/sampa/ (accessed Jan 8, 2020).

[B67] Seiger-Gardner L. Schwartz R. G. (2008). Lexical access in children with and without specific language impairment: a cross-modal picture-word interference study. *Int. J. Lang. Commun. Disord.* 43 528–551. 10.1080/13682820701768581 22612630

[B68] Senft V. Stewart T. C. Bekolay T. Eliasmith C. Kröger B. J. (2016). Reduction of dopamine in basal ganglia and its effects on syllable sequencing in speech: a computer simulation study. *Basal Gang.* 6 7–17. 10.1016/j.baga.2015.10.003

[B69] Sharma S. Aubin S. Eliasmith C. (2016). Large-scale cognitive model design using the Nengo neural simulator. *Biol. Inspir. Cogn. Architect.* 17 86–100. 10.1016/j.bica.2016.05.001

[B70] Stella M. Beckage N. M. Brede M. De Domenico M. (2018). Multiplex model of mental lexicon reveals explosive learning in humans. *Sci. Rep.* 8:2259. 10.1038/s41598-018-20730-5 PMC5797130 29396497

[B71] Stemberger J. P. (1985). An interactive activation model of language production. *Prog. Psychol. Lang.* 1 143–186.

[B72] Stewart T. C. (2012). *A Technical Overview Of The Neural Engineering Framework.* Waterloo, ON: University of Waterloo.

[B73] Stewart T. C. Bekolay T. Eliasmith C. (2012). Learning to select actions with spiking neurons in the basal ganglia. *Front. Neurosci.* 6:2 10.3389/fninf.2013.0002 PMC3269066 22319465

[B74] Stewart T. Choo F. X. Eliasmith C. (2012). Spaun: A perception-cognition-action model using spiking neurons. *Proc. Ann. Meet. Cogn. Sci. Soc.* 34 1018–1023.

[B75] Stewart T. C. Choo X. Eliasmith C. (2010a). “Dynamic behaviour of a spiking model of action selection in the basal ganglia,” in *Proceedings of the 10th International Conference On Cognitive Modeling*, Waterloo, ON.

[B76] Stewart T.C. Choo X. Eliasmith C. (2010b). “Symbolic reasoning in spiking neurons: a model of the cortex/basal ganglia/thalamus loop,” in *Proceedings of the Annual Meeting of the Cognitive Science Society*, New York, NY.

[B77] Stewart T. C. Eliasmith C. (2014). Large-scale synthesis of functional spiking neural circuits. *Proc. IEEE* 102 881–898. 10.1109/jproc.2014.2306061

[B78] Stewart T. C. Thorgeirsson S. Eliasmith C. (2018). Supervised learning of action selection incognitive spiking neuron models. *Proc. Ann. Meet. Cogn. Sci. Soc.* 40 1086–1091.

[B79] Stille C. M. Bekolay T. Blouw P. Kröger B. J. (2019). Natural language processing in large-scale neural models for medical screenings. *Front. Robot. AI* 6:62 10.3389/fninf.2013.00062 PMC7805752 33501077

[B80] Stimley M. A. Noll J. D. (1991). The effects of semantic and phonemic prestimulation cues on picture naming in aphasia. *Brain Lang.* 41 496–509. 10.1016/0093-934x(91)90170-6 1723331

[B81] Van Hees S. Angwin A. McMahon K. Copland D. (2013). A comparison of semantic feature analysis and phonological components analysis for the treatment of naming impairments in aphasia. *Neuropsychol. Rehabil.* 23 102–132. 10.1080/09602011.2012.726201 23098246

[B82] Velez M. Schwartz R. G. (2010). Spoken word recognition in school-age children with SLI: Semantic, phonological, and repetition priming. *J. Speech Lang. Hear. Res.* 53 1616–1628. 10.1044/1092-4388(2010/09-0042) 20798326

[B83] Vitevitch M. S. Chan K. Y. Roodenrys S. (2012). Complex network structure influences processing in long-term and short-term memory. *J. Mem. Lang.* 67 30–44. 10.1016/j.jml.2012.02.008 22745522 PMC3381451

[B84] Voelker A. R. Crawford E. Eliasmith C. (2014). Learning large-scale heteroassociative memories in spiking neurons. *Unconvent. Computat. Nat. Comput.* 7:2014.

[B85] Wambaugh J. (2003). A comparison of the relative effects of phonologic and semantic cueing treatments. *Aphasiology* 17 433–441. 10.1080/02687030344000085

[B86] Wambaugh J. L. Linebaugh C. W. Doyle P. J. Martinez A. L. Kalinyak-Fliszar M. Spencer K. A. (2001). Effects of two cueing treatments on lexical retrieval in aphasic speakers with different levels of deficit. *Aphasiology* 15 933–950. 10.1080/02687040143000302

